# Are victims of bullying primarily social outcasts? Person‐group dissimilarities in relational, socio‐behavioral, and physical characteristics as predictors of victimization

**DOI:** 10.1111/cdev.13772

**Published:** 2022-04-20

**Authors:** Tessa M. L. Kaufman, Lydia Laninga‐Wijnen, Gerine M. A. Lodder

**Affiliations:** ^1^ 3647 ICS & Sociology University of Groningen Groningen The Netherlands; ^2^ 8125 Pedagogy & Educational Sciences Utrecht University Utrecht The Netherlands; ^3^ 7899 Developmental Psychology Tilburg University Tilburg The Netherlands

## Abstract

Existing literature has mostly explained the occurrence of bullying victimization by individual socioemotional maladjustment. Instead, this study tested the person‐group dissimilarity model (Wright et al., *Journal of Personality and Social Psychology*, 50: 523–536, 1986) by examining whether individuals’ deviation from developmentally important (relational, socio‐behavioral, and physical) descriptive classroom norms predicted victimization. Adolescents (*N* = 1267, *k* = 56 classrooms; *M*_age_ = 13.2; 48.7% boys; 83.4% Dutch) provided self‐reported and peer‐nomination data throughout one school year (three timepoints). Results from group actor–partner interdependence models indicated that more person‐group dissimilarity in relational characteristics (fewer friendships; incidence rate ratios [IRR]_T2_ = 0.28, IRR_T3_ = 0.16, fewer social media connections; IRR_T3_ = 0.13) and, particularly, lower disruptive behaviors (IRR_T2_ = 0.35, IRR_T3_ = 0.26) predicted victimization throughout the school year.

**Abbreviations**
AIC: Akaike's information criterion
BIC: Bayesian information criterion
FDR: false discovery rate
GAPIM: group actor–partner interdependence model
IRR: incidence rate ratio
*SD*
: standard deviation
SNARE: Social Network Analysis of Risk behavior in Early adolescence

In early adolescence, desires to fit in and belong to the peer group are heightened, and being the target of peer victimization takes a substantial social and emotional toll (e.g., McDougall & Vaillancourt, 2015). A myriad of studies have aimed to identify which individual characteristics, especially socio‐behavioral maladjustment, place adolescents at risk for victimization (Zych, Farrington, et al., 2020). However, a sole focus on individual characteristics is insufficient to consider the fundamentally social function that bullying serves in the peer group; furthermore, it does not explain why, for many youths, bullying stops when they are placed in a different context (e.g., Bowes et al., 2013). Therefore, it is of vital importance to take a contextual “individual by group” approach in determining what types of adolescents within a particular context are more likely to be victimized. One commonly raised assumption, that has received surprisingly little attention within longitudinal empirical research to date (Boele et al., 2017) is that *deviating from peer norms* would predict victimization (Juvonen & Gross, 2005; Wright et al., 1986). According to the person‐group dissimilarity model (Wright et al., 1986), deviating from the descriptive group norm—defined as the average levels of a certain behavior or characteristic in a group—can make individuals become “social outcasts” and, therefore, rejected by the group; potentially, they can even become victims of bullying. In particular, this may be the case for developmentally salient characteristics such as forming stable social relationships and pubertal maturation.

Addressing this gap, this study examines (1) whether dissimilarity between individual characteristics and descriptive norms in the classroom predicts victimization and (2) whether this effect is larger when the norm is more homogeneous, that is, when others’ levels of the characteristic are closer to each other. We focus on early adolescence, the period between 10 and 14 years of age, during which peer groups in general and conformity to “fit in” take on heightened importance (Kindermann & Gest, 2018; Laursen & Veenstra, 2021). Relatedly, involvement in victimization and bullying also peaks in early adolescence (Zych, Ttofi, et al., 2020). We focus on characteristics that are important in this developmental phase and, therefore, determine “fitting in”: relational characteristics (friendships, social media connections; Laninga‐Wijnen & Veenstra, in press), socio‐behavioral characteristics (social anxiety, disruptive behaviors; Bass et al., 2016), and physical characteristics (pubertal development; Petersen et al., 1988). The classroom is a relevant context to examine the possible effects of person‐group dissimilarity on bullying because this is a peer group in which adolescents spend much of their time.

## Person‐group dissimilarity model

The person‐group dissimilarity model (Wright et al., 1986) builds on one of the most firmly established phenomena in social psychology: associations between similarity and liking, and dissimilarity and disliking—which are observed throughout all stages of development, including infancy (Sanefuji et al., 2006). Individuals consider those who are like them as belonging to their own group, or the “in‐group,” and those who are dissimilar to them as the “out‐group.” The person‐group dissimilarity model operationalizes similarity in valued and shared characteristics among group members as (descriptive) group norms, and postulates that those who deviate from salient norms will be rejected in an attempt to protect the norm and the social identity of the group (Hogg, 2018). A norm provides guidelines for how one should behave: It is a code of conduct that is essential for the smooth functioning of a peer group. For social norms to be effective means for maintaining social order, social safety, and well‐being, enforcement mechanisms include sanctioning any deviation from this norm with rejection or perhaps even bullying (Bass et al., 2021; Kindermann & Gest, 2018; Laursen & Veenstra, 2021). Thus, while previous research focused mostly on individual risks for victimization (e.g., Zych, Farrington, et al., 2020), it seems that the very same individual characteristic can be socially approved of and result in social acceptance in one group but can be socially disapproved of and result in rejection in another group. Empirical research provides some support for the notion that deviating from group norms relates to being rejected by peers. For example, cross‐sectional studies demonstrated that pre‐adolescent youths who were more aggressive and more withdrawn than the average in a group were rejected by more others in the group (Boor‐Klip et al., 2017; Chang, 2004; Velásquez et al., 2016).

Person‐group dissimilarity is likely associated not only with rejection but even with victimization through bullying (Juvonen & Gross, 2005), which is a more extreme, detrimental, and conscious phenomenon than rejection. Bullying is repeated, goal‐directed behavior that harms another individual in the context of a power imbalance and can take physical, verbal, relational (i.e., social exclusion and rumor spreading), and online forms (Olweus, 1993). Group members might not only dislike or reject individuals who do not fit in, they might even go as far as actively excluding or harassing norm deviants through bullying. They might do so not only to preserve the group norm, to punish those who break the “code of conduct” (Kindermann & Gest, 2018; Laursen & Veenstra, 2021; Thörnberg & Delby, 2019), but also because these rejected adolescents are efficient targets for bullies who aim to improve their own social status through bullying. Indeed, prior studies of young adolescents have indicated that bullies strategically target individuals who are the least likely to receive support from group members, thereby minimizing the costs of their negative actions in terms of a loss of affection or status (de Vries et al., 2021). Individuals who are dissimilar to many peers already occupy a rejected and socially isolated position, and bystanding witnesses are less likely to convey their disapproval when adolescents who are dissimilar to them or to the group to which they belong are bullied (Huitsing et al., 2014). Moreover, these adolescents who deviate from group norms may also have fewer peers who stand up for them because they have less in common with their peers: Similarity breeds connection with others and young adolescents help those to whom they are similar and do not help those to whom they are dissimilar (van Rijsewijk et al., 2016). Thus, norm‐deviating adolescents are easy targets to bully.

Tentative suggestions have already been made that are in line with this expectation that not only individual characteristics, but also their interactions with group norms contribute to the risk of becoming a victim of bullying. Cross‐sectional research indicates that young adolescents who are less sociable (Bass et al., 2016) and have dissimilar personality characteristics to those of the peer group (Boele et al., 2017) are victimized more frequently. More indirect evidence can be found in research on bias‐based bullying, revealing that minority groups such as ethnic, gender (e.g., non‐binary, transgender), sexual, or other minority youths or those with disabilities are at risk of being victimized (e.g., Russell et al., 2012). In qualitative research, young adolescents described deviance as the most common reason for bullying: They explained that they or their peers were bullied because they were “odd” or “different” (e.g., Thörnberg & Delby, 2019).

Descriptive norms, which indicate how common certain characteristics are in a group context, may be especially meaningful determinants of victimization when peers are *homogeneous* in the characteristics that make up these norms (Garcia et al., 2015). For example, if a few students in the classroom score highly for disruptive behaviors while the remainder of the students does not, this may render a relatively high descriptive norm for acting disruptively. However, this norm does not consider the strong variations among students in these behaviors within this classroom. This is important, because group identification and cohesion may be lower in heterogeneous classrooms than in homogeneous classrooms (Hogg, 1993). Therefore, in heterogeneous classrooms, norms—and conformity to them—might be less emphasized and less important (Juvonen, 2018). However, variation around the norms has been largely neglected in prior research on person‐group dissimilarity.

Altogether, despite the valuable insights into how groups reject adolescents who deviate from group norms, a vital next step is to test whether these patterns also translate into bullying, especially in classrooms with homogeneous norms. In doing so, several methodological issues of previous studies should be addressed. First, prior studies included the individual's score as part of the group's norm score, which, by default, makes it difficult to test person‐group dissimilarity. Second, prior research did not consider the potentially important role of homogeneity of norms. Both shortcomings can be addressed by using the group actor–partner interdependence model (GAPIM; Garcia et al., 2015), but these methods were scarcely used because they required updating for the use of *continuous* instead of dummy variables as predictors. These updates, however, have been developed and adopted for the purpose of the current study (Kenny, 2021). Finally, most studies have used cross‐sectional data, making it impossible to draw conclusions about the direction of effects. For example, victimization might predict relational or socio‐behavioral deviation from the norm instead of vice versa (Tieskens et al., 2019), because victims may be less well‐attached to the group and less motivated to conform to its norms. Longitudinal research on person‐group dissimilarity also helps to understand how quickly these processes unfold and continue, which determines the severity of practical implications of norm deviation. Deviating from norms at the beginning of the school year, when the classroom hierarchy is formed, may predict victimization in the short term, but that victimization may also last throughout the school year. This is especially true in contexts where classrooms remain similar throughout a school year, such as in the sample in the current Dutch study.

Addressing these concerns, our central research question was to examine the effects of deviance from descriptive group norms on young adolescents’ victimization experiences throughout the school year (Juvonen & Gross, 2005; Wright et al., 1986), while excluding the individual adolescent from the calculation of the group norm and considering norm homogeneity. We expected that more deviation from homogeneous norms predicted victimization. Specifically, this means that, when comparing adolescents within the same classroom regarding the extent to which they deviate from a salient norm in their classroom, adolescents who score *very* different from the norm are likely worse off than those who differ *slightly* from the classroom norm. This may particularly occur in the case of norm homogeneity.

## Relational, socio‐behavioral, and physical characteristics

Although previous studies focused mostly on norm deviation in terms of behavioral characteristics such as externalizing behaviors and withdrawal, expanding our knowledge on person‐group dissimilarity requires including a broader range of characteristics that adolescents consider to be important. From a developmental perspective, adolescents will attach value to characteristics that relate to their developmental goals, and, thus, reject individuals who deviate with respect to these characteristics (“maturational deviance” hypothesis; Petersen et al., 1988). We focus on three types of characteristics that are developmentally relevant to adolescents: relational, socio‐behavioral, and physical (pubertal development) characteristics.

Notably, the person‐group dissimilarity model postulates that rejection results from deviation from group norms in any direction, regardless of whether individuals score either lower or higher than salient norms. Alternatively, when we consider that bullying is often a goal‐directed behavior, adolescents might not be at risk for victimization when they deviate from the norm in a direction that clearly increases their access to social resources (e.g., among those who have *more* friendships than the norm). These adolescents still stand out from norms, but bullies would not dare to target them because doing so would jeopardize their own status too much. Consequently, deviation may be disadvantageous except if this deviation constitutes increased social resources and, therefore, reduces risk. Accordingly, some of our hypotheses explained below are directional in nature.

### Relational characteristics

A fundamental social goal in early adolescence, in particular after a transition to a new peer context such as secondary school, is to form stable, higher‐quality peer relationships (e.g., Laninga‐Wijnen & Veenstra, in press). Friendships are clear indicators of achieving this goal, as friendships are often more stable and intimate than other peer relationships. Furthermore, *online* peer relationships are also crucial to adolescents today. Social media have become increasingly central to adolescents’ lives and have become part of their peer culture (Nesi et al., 2018). Adolescents can count and compare their online friend lists or “followers” as indicators of status, and prior research has demonstrated that young people are significantly aware of their own list of followers and compare it to the lists of others (Nesi et al., 2018). This demonstrates that adolescents pay great attention to their own and their peers’ (online) relational characteristics, and, thus, to the group norms for these relational characteristics. Additionally, individuals who deviate more from this norm than others, may be more likely to be victimized.

However, for these relational characteristics, it can be expected that deviating from the norm only by having *fewer* (online) peer relationships may be risky. Young adolescents who have *more* peer relationships than the norm will also have more social support, which reduces their risk for victimization (van Rijsewijk et al., 2016). Therefore, we expected that more dissimilarity to the descriptive classroom norms in these relational characteristics would predict more individual victimization throughout the school year only if individuals’ number of friendships (H1a) or social media connections (H1b) were *lower* than the classroom norm.

### Socio‐behavioral characteristics

To satisfy the increasing need to fit in with a peer group during adolescence, exhibiting socially appropriate behaviors seems vital. Important determinants of social interactions can be classified into internalizing and externalizing dimensions, so it is relevant to consider deviation from the norm regarding characteristics that relate to both dimensions. We focus on socially anxious affect, and on disruptive behavior (being cheeky, disobedient, starting fights) as a type of externalizing behavior. Individuals who deviate more from norms related to these internalizing and externalizing dimensions will clearly stand out in social interactions, potentially resulting in victimization (Bass et al., 2021; Wright et al., 1986). Regarding the internalizing dimension, social anxiety is not a behavior in itself, but socially anxious affect relates strongly to internalizing behaviors, such as withdrawal, which are noticed by peers (Barzeva et al., 2020). In a classroom where social anxiety is, on average, low, peers will approach each other frequently, and the shyer and more timid individuals may stand out. However, for individuals who are *less* anxious than the norm, deviation might not be a risk, even though their behaviors are different from those of most peers. Bullies might not want to risk their status by targeting adolescents who are relatively low in social anxiety, because these adolescents often have more social resources than their peers do (de Lijster et al., 2018).

Regarding the externalizing dimension, in a classroom where it is normative to act disruptively—for example, by being cheeky to teachers—such behavior might be considered the “expected” way to interact, and the individuals who do not join in with such behaviors stand out. In contrast, in groups whose members behave well and are obedient, the individuals who act relatively disruptively will be considered deviant. The more they deviate from the behavioral norm, the more they might be victimized (Bass et al., 2021; Wright et al., 1986).

In sum, for social anxiety, we hypothesized that more dissimilarity in individual adolescents’ anxiety (H2a) to the descriptive classroom norm for anxiety predicts more individual victimization only if individuals’ anxiety would be *higher* than the classroom norm. For disruptive behaviors, we hypothesized that more dissimilarity in individual adolescents’ disruptive behaviors (H2b) to the descriptive classroom norm for disruptive behaviors would predict more individual victimization regardless of whether adolescents acted *less* or *more* disruptive than the classroom norm.

### Physical characteristics

Sexual maturity is another central milestone for adolescents. The first landmark is the onset of puberty; hormonal changes set the stage for a range of different physical changes, behaviors, and interests, such as increases in risk behavior and sexual behavior (Baams et al., 2015; Harden et al., 2018). As such, adolescents who deviate from their classmates in the onset of puberty, either earlier or later, likely stand out from what is normative (Petersen et al., 1988), and they may be at increased risk for being victimized as a result. Research provides some tentative support for this by indicating that adolescents who experience an early onset of puberty compared to the norm are more likely to be victimized by peers (Haynie & Piquero, 2006; Skoog & Kapetanovic, 2021; Troop‐Gordon, 2017). Conversely, in a group in which peers exhibit more advanced pubertal maturation, those who do not yet show the visible signs of puberty (e.g., no pubic hair, no voice changes, or for girls no breast development) or who do not yet indicate interests in sex(uality) that are triggered by puberty might be considered to be childish and uncool.

Pubertal development is caused by sex‐specific hormones and, therefore, the related changes are sex‐specific (e.g., Baams et al., 2015). Consequently, measures and group norms are also sex‐specific, and “fitting in” is determined by similarity only to same‐gender peers. Still, within each sex group, the effects of person‐group dissimilarity are expected to occur in a like manner (Skoog & Kapetanovic, 2021). In sum, we hypothesized that more dissimilarity in individual boys’ and girls’ pubertal development (H3) to the descriptive classroom norm for pubertal development—regardless of whether this would be in terms of being *less* or *more* advanced—would predict more individual victimization.

## Current study

Our study is one of the first that longitudinally tested theoretical suggestions (Juvonen & Gross, 2005) based on the person‐group dissimilarity model (Wright et al., 1986) that young adolescents who deviate from (homogeneous) group norms are at higher risk of being victimized. In doing so, we expanded current knowledge by (1) broadening the focus from social preference and rejection to *victimization*, (2) considering *homogeneity* of the norms, (3) including a *broader range* of developmentally relevant characteristics, (4) including the hypothesized *directions* of effects in our analyses, and (5) applying an advanced statistical technique that excluded individual scores from *norm calculation*, using (6) *longitudinal* data across varying time spans.

We addressed the methodological concerns of prior research by using the GAPIM (Garcia et al., 2015) model and the recently developed formulas for the use of continuous measures (Kenny, 2021). We estimated the extent to which more individual dissimilarity from the classroom norm at the start of the school year predicted victimization at two later time points during the school year (3 and 6 months after the start, respectively) while the effect of individual and group averages of these characteristics on victimization were taken into account. This enabled us to understand how long it took for the potential effects to unfold and to examine whether these effects lasted.

First, we expected that greater dissimilarity between individual characteristics and classroom norms predicted higher levels of victimization. Focusing on relational, socio‐behavioral and physical characteristics, we expected that more dissimilarity to the descriptive classroom norm in terms of friendships (H1a), social media connections (H1b), social anxiety (H2a), disruptive behaviors (H2b), and pubertal development (H3a, boys; H3b, girls) predicted more victimization. For H1a, H1b, and H2a, our hypotheses were directional: We expected that higher levels of dissimilarity predicted increased victimization only among adolescents who had *fewer* friendships or social media connections, or *more* social anxiety than the norm. For H2b and H3, hypotheses were not directional: We expected that greater deviation in terms of both lower and higher levels of disruptive behaviors, and both earlier and later pubertal development than the classroom average would predict higher levels of victimization. Second, we expected that all person‐group dissimilarity effects would be stronger in classrooms where peers were relatively homogeneous in their characteristics, than in classrooms where peers varied more significantly in their characteristics (heterogeneous classrooms). These hypotheses were pre‐registered (https://aspredicted.org/sp5q2.pdf).

## METHOD

### Procedures and participants

The data used in this research stem from the Social Network Analysis of Risk behavior in Early adolescence (SNARE) study, a prospective cohort study on the social development of young adolescents, conducted at two secondary schools in the middle and northern parts of the Netherlands (Dijkstra et al., 2015). Participants were recruited in their first or second grade of secondary school (US grades 7–8, school years 2011–2012). Participants and their parents received an information letter and provided passive consent (refusal rate = 0.1% in the current sample). If parents did not want their child to participate, they were asked to send a reply card or email within 2 weeks. Adolescents could opt out from participation at any time. The study was approved by the Ethical Internal Review Board of one of the participating universities (Utrecht University).

We focus on the first three waves of the SNARE study, which were conducted in October (T1) of the first or second year of secondary school and December (T2) and March (T3) of the same school year. The sample consisted of 1267 adolescents (*N* = 56 classrooms; 48.7% boys, *M*_ageT1_ = 13.2) who participated in at least one of the three waves (average N of students per classroom = 23.2, min = 12; max = 29); for details on inclusion criteria see Supporting Information 1. Participants reported ethnic backgrounds of Dutch (83.4%), Turkish (1.4%), Moroccan (2.5%), Surinamese (1.6%), Indonesian (0.9%), Netherlands Antillean and Aruban (0.5%) or other (9.8%) ethnic background. In the Netherlands, the classroom composition for adolescents in these grades remains the same throughout the school year and differs across school years.

### Measures

*Victimization (Bully‐reported: T1–T3)* was assessed as the incoming number of peer nominations based on one question: *Whom do you bully?* We used bully‐reported victimization because this seems to be a stronger indicator of whether someone fits with the peer group than the subjective experience of being victimized (Boele et al., 2017). However, in our sensitivity analyses, we used a victim‐reported peer nomination measure: the outgoing number of possible sent nominations based on the question *Who bullies you?*.

*Friendships* (T1) were assessed as the received number of peer nominations based on one question: *Which classmates are your best friends?* We divided the number of nominations by the possible number of nominators in the classroom and the scores thus represented the proportion of classmates who had nominated an individual adolescent on a theoretical range of 0–1 (Lease et al., 2002; Logis et al., 2013).

*Social media connections* (T1) were assessed as participants’ self‐reported number of followers on a popular social networking website, Hyves, in the Netherlands at the time of survey administration. The original answers ranged from 0 to 1000 friends. However, the answers were classified into four categories based on the 25%, 50%, and 75% percentiles in the data (Q1 = 103, Q2 = 210, Q3 = 328) because the difference between 750 and 1000 followers might be less meaningful than the difference between, for example, 250 and 500 followers. Common experiences indicated by percentiles were assumed to be more meaningful indicators of what was normative.

*Social anxiety* (T1) was measured as the mean of eight self‐reported items from the Social Phobia Screening Questionnaire on how much fear participants generally experience in social situations (example item: “*speaking in front of the class*,” 1 = no fear to 5 = much fear; Gren‐Landell et al., 2009). This variable was assessed in September instead of October (*α* = .80).

*Disruptive behaviors* (T1) were measured as the mean of three incoming peer nomination questions. Peers could nominate each other in the following questions: *Who are regularly cheeky to teachers?*, *Who sometimes fight and*/*or pick a quarrel with you?*, and *Who break the rules (e*.*g*., *steal something or demolish a bus shelter)?* Participants could nominate an unlimited number of peers. We divided the number of received nominations by the number of peers. The reliability was below conventional standards (*α* = .58), perhaps because of the low number of items (proportion scores). Intercorrelations among the three items were moderate to strong (*r* = .35 to *r* = .62). Separate analyses with each item indicated findings in a similar direction as the main analyses using the composite (see Supporting Information 2).

*Pubertal development* (T1) was assessed as the mean of four (boys) or five (girls) items on physical changes related to pubertal development from the Pubertal Development Scale (Petersen et al., 1988): *How is your body growth in length (so*‐*called growth spurt)?*, *How is the growth of your body hair* (*e*.*g*., *armpit hair*, *pubic hair)?*, *How are the changes in your skin (greasy skin*, *pimples)?*, for boys *Has your voice changed (lower)?* and for girls *Have your breasts already started growing?* (all four: 1 = not changes to 4 = changes already past). Furthermore, girls were asked a fifth question, *Are you already menstruating?”* (0 = no, 1 = yes; 1 was recoded into 4, see Petersen et al., 1988). The measure was computed separately for boys (*α* = .76) and girls (*α* = .70), and the classroom norm was calculated among same‐gender peers only.

### Analytic strategy

We tested our hypothesis using the GAPIM in Stata 16 SE. We conducted Poisson mixed regression (confirmatory) analyses because the victimization measure was a count variable reflecting one's number of incoming nominations. To test our hypotheses, we examined and compared three sub‐models: (1) the *complete model*, which estimated the effect of person‐group similarity on victimization, while controlling for homogeneity of the norm, (2) the more parsimonious *person*‐*fit* model (Garcia et al., 2015), which did not control for homogeneity of the norm, and (3) the *contrast interaction* model, which assessed the effect of the person's similarity to others relative to the similarity of everyone else within a particular group (Garcia et al., 2015). We describe each submodel below.

#### The three GAPIM models: Complete, person‐fit, and contrast interaction models

##### Complete model

The *complete GAPIM model* included four variables as predictors: *x*, *x′*, *i*, and *i′*. The variable *x* represents individual scores on the predictor, such as the individual's average levels of disruptive behavior. The variable *x′* represents the average levels of this variable at the classroom level. The *x′* variable was calculated as the average score of the other *n* − 1 group members (classmates).

The third variable, the person‐group similarity score *i*, reflects the extent to which an individual's score is similar to the group's score on this characteristic, for example, the extent to which a participant's own level of disruptiveness is similar to the descriptive classroom norm for disruptiveness. The focus in this study is on the effect of this variable on victimization, because it represents the person‐group similarity effect. To calculate the *i* term, the square root of the absolute difference between the person and others’ (*x′*) scores was calculated. From this value, 1 was subtracted and then multiplied by –1, so that the absolute difference did not range from 0 to 2 but could (maximally) range from –1 to 1: where –1 reflects maximal dissimilarity and 1 reflects maximal similarity (Garcia et al., 2015). By taking the square root, the measure reflects the “distance” between *x* and *x′*. Positive values of *i* represent more similarity, while negative values represent more dissimilarity. Consequently, in the regression model, significant positive effects of *i* suggest that the more *similar* an individual was to their peers, the higher their levels of victimization were. A significant negative effect of *i* suggests that the more *dissimilar* an individual was to their peers, the higher their levels of victimization were.

The last variable of the complete GAPIM model is the group‐group similarity score *i′*. This term represents the average similarity of the scores of all possible pairs of others in the group (e.g., the similarity between each classmate's level of disruptiveness and the classroom‐average level of disruptiveness), so, homogeneity. The *i′* term is calculated as the square root of the average absolute value of the difference between all pairs excluding the person from which 1 is subtracted and multiplied by –1.

##### Person‐fit model and contrast interaction model

Besides the complete model, two alternative models were tested for each outcome of interest. The more parsimonious, *person*‐*fit model* excludes the effects of *i′* (group‐group similarity) on victimization (thus, included only *x*, *x′*, and *i*) when *x′* did not improve the model. Furthermore, the *contrast interaction model*, which assessed the effect of the person's similarity to others relative to the similarity of everyone else (Garcia et al., 2015), included an additional interaction effect to the complete model: the product of *i* × *i′*. This model, therefore, tests a three‐way interaction effect; whether the effect of person‐group similarity on victimization depends upon the extent to which group members are homogeneous in these characteristics.

Notably, the direct effects of individual and group scores (*x* and *x′*) on victimization were not interpretable in the GAPIM models because they were conditional on the presence of *i*. To reveal how youths’ individual and classroom scores for the characteristics were related to individual victimization, we additionally reported on models that omitted *i* or *i′* (see Supporting Information 3).

#### Testing directional hypotheses

For the models for which we had hypotheses in one specific direction, we performed directional analyses. This means that we first computed the *x′* term in the complete classroom and then selected the subsamples that were similar to or had *fewer* friends or social media connections than the norm (H1a, H1b: *x* ≤ *x′*), or that were similar to or *more* socially anxious than the norm (H2a: *x* ≥ *x′*), on which analyses were performed. The complete directional models only included *x*, *i*, and *i′* when the *x′* term did not improve the model fit, and made the model unnecessarily complex because significant variation in *x′* was already captured by the selection of subsamples.

#### Model selection and evaluation

##### Fit Indices

To determine the best‐fitting submodel (the complete, person‐fit, or contrast interaction model), the following criteria were used (Garcia et al., 2015): (1) the best submodel should fit at least as well as the complete model in terms of at least lower Akaike's information criterion (AIC) and/or Bayesian information criterion (BIC). Furthermore, (2) the effect of the term computed and added into the submodel should be statistically significant, and lastly, (3) it should be the best fitting submodel with statistically significant effects. For example, if the contrast interaction model fits best but does not exhibit additional significant effects compared with the complete or person‐fit model, it is not the final model to be chosen. When model fits were close to each other (Δ <10 points for both AIC and BIC; Burnham & Anderson, 2004) and included significant effects, we reported both. We did not report on the person‐fit model when the complete model (extending the person‐fit model with one covariate) fitted as well and included a significant group‐group (*i′*) effect.

##### Follow‐up analyses

When a significant person‐group dissimilarity effect was detected in a (non‐directional) complete or person‐fit model, we conducted follow‐up analyses to interpret the direction of effects, for example, to identify whether greater norm deviation among those who acted either *less* or *more* disruptively than the norm, predicted victimization. Like the main analyses for directional hypotheses, we estimated the model separately in subsamples with individuals ≤ versus > the descriptive norm. We evaluated the *i* effect to determine whether there was a person‐group similarity effect.

In follow‐up analyses of the contrast interaction models (with the term *i* × *i′*), we estimated the previous follow‐up analyses additionally among two subsamples that differed in the extent to which *others* were homogeneous (mean‐split of *i′*: subsamples that scored ≤ versus > than the grand mean for *i′*). In other words, we estimated the effects of person‐group similarity on victimization among four subsamples of (1) *x* ≤ *x′*, (2) *x* > *x′*, in groups that were (3) homogeneous (> grand‐mean of *i′*) and (4) heterogeneous (≤ grand‐mean of *i′*).

##### Incidence rate ratios

Regarding effect sizes, in addition to regression coefficients in logged form, we show the incidence rate ratio (IRR) of the person‐group similarity effects, which are estimated rate ratios for a 1‐unit increase in the predictor. The expected count (number of bullies) is multiplied by a factor of the IRR when the predictor increases by 1 unit, while holding all other covariates constant. To improve the interpretation of the IRR, we converted the IRR so that it reflected changes in standard deviations (*SD*s) instead of units. For example, IRR = 0.28 (*SD* = .21) means that a decrease of 1 *SD* in similarity predicted ([1 − 0.28] × 0.21=) a 15% increase in victimization. To reduce the risk for false discovery rates (FDRs) in regression analyses with five predictors, we used an FDR‐controlling procedure when determining statistical significance (Benjamini & Hochberg, 1995). For more details on the calculation of the terms, estimation, and the FDR method, see Supporting Information 1.

##### Covariates and sensitivity analyses

We controlled for the main effects of participants’ victimization at T1, age, gender, grade, and classroom size. We tested whether the results differed when using a self‐reported nomination measure of victimization (Supporting Information 4), without the inclusion of covariates (Supporting Information 5) or correcting outliers (Supporting Information 6) and tested gender moderation (Supporting Information 7). Additionally, we reported traditional multilevel models (Supporting Information 8), and friendship models with a measure of reciprocal, instead of received nominations for, friendships (Supporting Information 9). Finally, we reported directional models for *all* characteristics, so in addition to the hypothesized ones (Supporting Information 10), and reported results based on the more restrictive product method (*x* × *x′*) rather than the difference method to calculate the person‐group similarity term *i* (Supporting Information 11).

## RESULTS

Table 1 indicates the Pearson correlations and descriptive statistics. Victimization at different points in time was significantly yet weakly correlated with more disruptive behaviors and fewer friendships (all waves) and lower social media connectedness (T1).

**TABLE 1:** Pairwise Pearson correlations and descriptive statistics across all variables

Variables	1.	2.	3.	4.	5.	6.	7.	*M* (*SD*)
1. Victimization T1	—							.21 (.60)
2. Victimization T2	.38**	—						.22 (.62)
3. Victimization T3	.38**	.39**	—					.20 (.56)
4. Social anxiety T1	−.02	.02	.00	—				−.68 (.26)
5. Disruptive behaviors T1	.10**	.06*	.09**	−.24**	—			−.83 (.27)
6. Friendships T1	−.20**	−.23**	−.16**	−.09**	.04	—		−.35 (.34)
7. Social media conn. T2^a^	−.10**	−.03	.00	−.13**	.27**	.21**	—	−.01 (.74)
8. Boys’ pubertal dev. T1	.02	.04	−.01	−.09	.23**	−.04	.14**	−.24 (.44)
9. Girls’ pubertal dev. T1	.05	.07	.01	−.03	.12**	−.06	.15**	−.02 (.44)

^a^ Social media connectedness was measured only at T2.

* *p* < .05.

** *p *< .001.

### Results of GAPIM analyses

#### Descriptive analyses

First, for descriptive purposes, we estimated regression models that only included the person and group scores (Supporting Information 3), and no similarity terms. Predictors for victimization were having fewer friends and being in a classroom with a higher average for friendships (both short‐ and long‐term effects) or social media connections, as well as acting more disruptively (long‐term effect). Finally, boys who reported *less* advanced individual pubertal development were victimized more in the long term, while girls who reported *more* advanced pubertal development, and girls in classrooms with *more* advanced pubertal development, on average, were victimized more in the short term. The other effects were not significant.

#### Group actor–partner interdependence models

Table 2 shows the model fit (AIC and BIC) of the different GAPIM submodels, and Table 3 shows the results for the best‐fitting model(s) based on the criteria as described in the analytic strategy section (fit and significant effects). We discuss the results for each characteristic separately, in both the short term (3 months: changes in T1–T2 victimization) and long term (throughout the school year: changes in T1–T3 victimization).

**TABLE 2:** Comparison of Poisson GAPIM submodels for individual‐group similarity effects on victimization

Predictor at baseline (T1: October)	T2						T3					
	Person‐fit		Complete		Contrast interaction		Person‐fit		Complete		Contrast interaction	
	AIC	BIC	AIC	BIC	AIC	BIC	AIC	BIC	AIC	BIC	AIC	BIC
Friendships	**782.4**	**826.5**	784.3	832.8	785.1	837.9	735.0	779.0	**731.6**	**778.0**	733.1	785.8
Social media connectedness	n/a	n/a	n/a	n/a	n/a	n/a	**616.9**	**661.0**	618.3	666.9	620.3	673.3
Social anxiety	**496.8**	**539.4**	495.5	542.3	497.4	548.5	**445.7**	**488.2**	447.6	494.3	449.1	500.1
Disruptive behaviors	1241.9	1298.4	**1239.7**	**1301.4**	**1226.1**	**1293.0**	**1174.7**	**1231.2**	1176.6	1238.3	1178.6	1245.4
Boys’ pubertal development	**690.2**	**734.3**	689.1	737.6	691.1	744.0	691.1	744.0	**627.0**	**675.5**	627.6	680.5
Girls’ pubertal development	**490.3**	**534.6**	492.1	540.8	491.7	544.9	**491.7**	**544.9**	537.0	585.6	538.9	592.0

Note

Numbers in bold refer to the final model(s), which were multiple models if ΔBIC <10 and an additional effect (of *i′* in the complete model, or of *i* × *i′* in the contrast interaction model) was observed.

Abbreviations: AIC, Akaike information criterion; BIC, Bayesian information criterion; GAPIM, group actor–partner interdependence model.

**TABLE 3:** GAPIM submodels: Poisson estimations of individual‐group similarity effects on victimization

Characteristics at baseline (T1: October)	T2		T3	
	*b*	95% CI	*b*	95% CI
Directional analyses^a^				
Friendships				
Person‐fit/complete model				
Person score *x*	−0.89	−1.79; 0.01	−0.13	−1.07; 0.80
Person‐group similarity *i*	−1.27	−2.45; −0.11	**−1.74**	−2.95; −0.54
Group‐group similarity *i′*			**2.74**	0.54; 4.94
Social media connectedness				
Person‐fit model				
Person score *x*	n/a	n/a	**0.92**	0.16; 1.68
Person‐group similarity *i*	n/a	n/a	**−2.03**	−3.44; −0.62
Social anxiety				
Person‐fit model				
Person score *x*	−0.22	−2.07; 1.63	0.10	−1.85; 2.04
Person‐group similarity *i*	−0.58	−3.49; 2.32	−0.62	−3.72; 2.48
Non‐directional analyses				
Disruptive behaviors				
Person‐fit/complete model				
Person score *x*	−0.55	−1.20; 0.10	**−0.58**	−1.09; −0.07
Group score *x′*	**2.88**	0.04; 5.72	−0.86	−2.53; 0.81
Person‐group similarity *i*	**−1.09**	−2.02; −0.15	**−1.42**	−2.17; −0.66
Group‐group similarity *i′*	**2.75**	0.12; 5.38		
Contrast interaction model				
Person score *x*	0.22	−0.57; 1.00		
Group score *x′*	2.55	−0.25; 5.35		
Person‐group similarity *i*	1.50	−0.12; 3.12		
Group‐group similarity *i′*	2.21	−0.39; 4.81		
Contrast interaction (*i* × *i′*	**−3.88**	−5.82; ‐1.95		
Pubertal development				
Boys				
Person‐fit/complete model				
Person score *x*	−0.09	−0.59; 0.41	**−0.73**	−1.29; −0.18
Group score *x′*	2.00	−0.45; 4.46	1.42	−1.02; 3.86
Person‐group similarity *i*	−0.47	−1.41; 0.48	−0.04	−1.16; 1.08
Group‐group similarity *i′*			**4.19**	1.53; 6.85
Girls				
Person‐fit model				
Person score *x*	0.60	−0.04; 1.24	−0.30	−0.89; 0.29
Group score *x′*	**3.15**	0.68; 5.63	−0.85	−3.30; 1.61
Person‐group similarity *i*	0.32	−.78; 1.45	−0.27	−1.43; 0.89

Note

Numbers in bold represent significant findings at the individual level.

Abbreviation: GAPIM, group actor–partner interdependence model.

^a^ Directional analyses were conducted by only estimating the effect for individuals who scored ≤(friendships, *N* = 609, social media connectedness, *N* = 634) or ≥(social anxiety; *N* = 522) classroom norm *x′*. The group score *x′* did not improve the model and was excluded for parsimony.

##### Relational characteristics

Regarding friendships, for the short‐term effects, the person‐fit model fitted the data best and the other models did not show significant group‐group similarity effects or a contrast interaction effect. The person‐fit model showed a significant negative person‐group similarity effect. The fewer friends an individual had compared to the norm, the more they were victimized in the short term. Based on the IRR (IRR = 0.28), with every *SD* decrease in similarity (*SD* of *i* = .20), victimization increased by 14% in the short‐term ([1–0.28] × 0.2 = 0.14).

For the long‐term effect, the complete model fitted best and demonstrated a significant person‐group similarity effect. Furthermore, the contrast interaction model did not reveal a significant effect. In the long term as well, the fewer friends an individual had compared to the norm, the more they were victimized (IRR = 0.16, 17% more victimization per *SD* decrease in similarity). This effect considered (but did not interact with: no contrast interaction effect) others’ homogeneity in terms of friendships, group‐group similarity *i′*, with more homogeneity predicting more victimization.

For social media connectedness, we could estimate only short‐term effects (of T2 on T3) at the end of the school year, because it was measured only at T2. Here, the person‐fit model suited the data best and the other models neither indicated significant group–group similarity effects nor a contrast interaction effect. The person‐fit model revealed a significant negative person‐group similarity effect: The fewer social media connections an individual had compared to the norm, the more victimized they were (IRR = 0.13, *SD* = .21, 18% more victimization per *SD* decrease in similarity).

Altogether, results supported H1a (friendships) and H1b (social media connections): The fewer friends or social media connections that individuals had compared to their classroom's norm, the more they were victimized 3 months later in the school year (friendships, social media connections) and 6 months later (friendships).

##### Socio‐behavioral characteristics

For anxiety, for both the short‐ and the long‐term effects, the person‐fit models suited the data best, but revealed no significant effects. The other models did not indicate significant group–group similarity effects or a contrast interaction effect as well. This means that being dissimilar from the norm in terms of being more anxious did not significantly predict victimization in this sample (H2a).

For disruptive behaviors, regarding the short‐term effect, both the complete and the contrast interaction model fitted the data best and included, respectively, significant group–group similarity and contrast interaction effects. The models were not substantially different from each other (ΔBIC <10) and therefore we reported both. The complete model revealed a significant negative person‐group similarity effect (IRR = 0.35). Being more dissimilar in individual disruptive behaviors to the classroom norm predicted more victimization, while considering others’ homogeneity in terms of disruptive behavior (group–group similarity *i′*). Follow‐up analyses showed that the *less* disruptively the individuals acted compared to the classroom norm, the more victimization these individuals experienced in the short term (*b*_i_ = −1.14, 95% CI = −2.09; −0.19, IRR = 0.32), an effect that was not observed for acting *more* disruptively than the norm (but *p* = .07; *b*_i_ = −1.75, 95% CI = −3.65; 0.15, IRR = 0.17).

The contrast interaction model additionally revealed that this effect interacted with the extent to which *others* were similar (*i* × *i′*). As expected, more dissimilarity among those who acted *less* disruptively than the norm especially predicted victimization in *more homogeneous* classrooms—in other words, those in which peers were similar in their higher levels of disruptive behaviors (*b*_i_ = −2.22, 95% CI = −3.57; −0.88, IRR = 0.11) and not dissimilar (*b*_i_ = 2.28, 95% CI = −0.58; 5.14, IRR = 9.80). There were no person‐group similarity effects of acting *more* disruptively in either homogeneous (*b*_i_ = −0.50, 95% CI = −4.24; 3.26, IRR = 0.61) or heterogeneous (*b*_i_ = 0.56, 95% CI = −2.36; 3.75, IRR = 1.75) classrooms, but these findings should be interpreted with caution considering the small number of victimized individuals in these contexts (*N* = 6 in the heterogeneous sample). Altogether, among those who acted less disruptively than the homogeneous norm, with every *SD* (=.15) decrease in similarity, their victimization increased by 13%.

Over the long term, the person‐fit model fitted best, and the other models neither exhibited significant group–group similarity effects, nor a contrast interaction effect. The model demonstrated a negative person‐group similarity effect (IRR = 0.26): more dissimilarity in terms of disruptive behaviors predicted more victimization. Directional follow‐up analyses indicated that, in contrast to the short‐term direction of effects, that similarity did not predict victimization among adolescents who acted *less* disruptively than the classroom norm (*b*_i_ = −0.34, 95% CI = −1.26; 0.57, IRR = 0.71) but it did predict more victimization among those who acted *more* disruptively than the classroom norm (*b*_i_ = −1.85, 95% CI = −3.60; −0.10, IRR = 0.16) with a 34% increase per *SD* decrease in similarity.

Altogether, more deviation from the classroom norm for disruptive behaviors predicted more victimization throughout the school year (H2). However, the direction of effects differed depending on the moment in the school year. The *less* disruptively individuals acted compared to the classroom norm, especially in more homogeneous classrooms, the more these individuals were victimized 3 months after the beginning of the school year. In contrast, 6 months after the beginning of the school year, it was especially risky to deviate more from the initial classroom norm in terms of acting *more* disruptively.

##### Physical characteristics

For pubertal development, both for boys and girls and both for the short‐ and long‐term effects models, the person‐fit models suited best but revealed no significant person‐group similarity effects. Additionally, the other models did not indicate significant effects either. Consequently, deviating from same‐gender classmates in one's stage of pubertal development thus did not predict victimization (H3). Homogeneity of the group norms did not contribute to the effects either, except that boys in more homogeneous classrooms (group–group similarity effect *i′*) were victimized more in the long term.

### Sensitivity analyses

We conducted various sensitivity analyses (see Supporting Information 4–11). First, sensitivity analyses using a self‐reported instead of bully‐reported measure of victimization complemented the main findings (Table 4, and, for details, see Supporting Information 4).

**TABLE 4:** GAPIM submodels: Poisson estimations of individual‐group (dis)similarity effects on self‐reported victimization

Characteristics at baseline (T1: October)	T2		T3	
	*b*	95% CI	*b*	95% CI
Directional analyses^a^				
Friendships				
Person‐fit model				
Person score *x*	**−1.45**	−2.54; −0.35	−0.60	−1.54; 0.34
Person‐group similarity *i*	−0.59	−2.04; 0.87	−0.16	−1.48; 1.17
Social media connectedness				
Person‐fit model				
Person score *x*	n/a	n/a	−0.09	−0.62; 0.60
Person‐group similarity *i*	n/a	n/a	0.58	−0.64; 1.80
Social anxiety				
Person‐fit model				
Person score *x*	**−1.58**	−2.99; −0.18	**−2.45**	−4.07; −0.83
Person‐group similarity *i*	**−3.32**	−5.62; −1.02	**−4.27**	−7.00; −1.55
Contrast interaction model				
Person score *x*	**−3.21**	−4.99; 1.43	−0.62	−2.53; 1.30
Person‐group similarity *i*	**−7.41**	−10.83; −4.00	−0.14	−3.81; 3.54
Group‐group similarity *i′*	−0.84	−4.03; 2.35	−3.35	−7.26; 0.55
Contrast interaction (*i* × *i′*)	**13.53**	5.55; 21.5	**−13.38**	−21.27; −5.50
Non‐directional analyses				
Disruptive behavior				
Person‐fit model				
Person score *x*	−0.25	−0.71; 0.21	0.38	−0.14; 0.90
Group score *x′*	−0.32	−1.89; 1.25	−0.06	−2.21; 2.09
Person‐group similarity *i*	**−1.08**	−1.75; −0.41	0.31	−0.43; 1.05
Pubertal development				
Boys				
Person‐fit/complete model				
Person score *x*	−0.02	−0.46; 0.42	−0.43	−0.99; 0.12
Group score *x′*	2.14	−0.38; 4.65	−0.48	−4.03; 3.07
Person‐group similarity *i*	0.02	−0.84; 0.87	0.52	−0.52; 1.56
Group‐group similarity *i′*	**3.61**	1.05; 6.18		
Contrast interaction model				
Person score *x*			−0.54	−1.11; 0.03
Group score *x′*			−0.07	−3.64; 3.32
Person‐group similarity *i*			**3.39**	0.90; 5.87
Group‐group similarity *i′*			**4.28**	0.67; 7.90
Contrast interaction (*i* × *i′*)			**−9.73**	−18.67; −0.78
Girls				
Person‐fit model				
Person score *x*	−0.33	−0.74; 0.08	−0.16	−0.61; 0.29
Group score *x′*	−0.92	−3.11; 1.27	0.03	−2.21; 2.27
Person‐group similarity *i*	−0.07	−0.85; 0.72	0.11	−0.71; 0.93

Note

Numbers in bold represent significant findings at the individual level.

Abbreviation: GAPIM, group actor–partner interdependence model.

^a^ Directional analyses were conducted by only estimating the effect for individuals who scored ≤(friendships, social media connectedness) or ≥(social anxiety) classroom norm *x′*. The group score *x′* did not improve the model and was excluded for parsimony.

In terms of relational characteristics, in contrast to the results for bully‐reported victimization, no person‐group similarity effects were observed; therefore, being more dissimilar to others in terms of friendships or social media connections did not predict victimization. Regarding social anxiety, however, additional and consistent effects were observed among those who were *more* socially anxious than the classroom norm, both in the short term and long term. Especially in *heterogenous* classrooms, being more dissimilar to the norm predicted more victimization among those who were more socially anxious than the classroom average, with 28%–29% increases in victimization per *SD* decrease in similarity. Regarding disruptive behavior, results showed negative person‐group similarity effects of disruptive behaviors in a similar way as the bully‐reported results did (yet only in the short term). The more individuals who acted less disruptively than the classroom norm deviated from the norm, the more they were victimized, with a 24% increase in victimization per *SD* decrease in similarity. We found no significant effects of pubertal development, in line with the analyses using the bully‐reported measure.

In addition to the sensitivity analyses on self‐reported victimization, the second (without covariates; Supporting Information 5) and third (without correction for outliers; Supporting Information 6) types of sensitivity analyses replicated the main analyses in demonstrating person‐group similarity effects for friendships, social media connectedness, and disruptive behaviors.

Fourth, no gender differences emerged (Supporting Information 7) except for the long‐term effects of disruptive behaviors. The person‐group dissimilarity effect of acting more disruptively than the norm found in the main analyses seemed slightly stronger for boys than for girls. However, this difference was based on non‐significant follow‐up results for girls despite a significant omnibus person‐group similarity effect among girls as well, and, consequently, this finding should be interpreted cautiously.

Fifth, we examined traditional multilevel models (Supporting Information 8) in addition to the GAPIM approach. The conclusions based on these traditional models were similar to the GAPIM findings by also highlighting person‐group dissimilarity in friendships and disruptive behaviors as predictors of victimization. For social media connectedness and social anxiety, mixed patterns were revealed. These partly divergent findings may be due to the substantially different way in which the traditional models test dissimilarity effects. They are non‐directional (e.g., also testing person × group interactions in classrooms with high‐anxiety norms), and limited to testing main effects across low‐ versus high‐average norm contexts, instead of directly testing the extent to which individuals deviated from classroom norms as done in the GAPIM analyses. Moreover, they do not test interactions with norm homogeneity.

Sixth, a measure of reciprocal instead of unilateral (those for which nominations were *received*) friendships demonstrated person‐group dissimilarity effects similar to the main analyses (see Supporting Information 9). The long‐term effect of friendship similarity was non‐significant, but an additional significant short‐term effect for *self*‐reported victimization as an outcome was observed, especially in more homogeneous classrooms. The fewer reciprocated friendships individuals had compared to the norm, the more victimized they were, especially when peers were more similar in their number of friendships.

Seventh, we conducted directional hypotheses for nonhypothesized directions of effects and nondirectional hypotheses (see Supporting Information 10). Results did not change the conclusions: There were no effects of person‐group similarity in the nonhypothesized directions, and no effects in directions that were not tested because of a lack of an omnibus effect of that characteristic. For example, there were no person‐group similarity effects among those who scored *lower* or *higher* than the descriptive pubertal development norm.

Eighth, a more stringent method to calculate similarity, namely, creating a product term (person score *x* × group score *x′*) supported the robustness of our findings (Supporting Information 11).

## DISCUSSION

This study examined whether “being different” can be a risk factor for victimization in early adolescence. In detail, we examined whether deviation from the descriptive classroom norm in terms of relational characteristics (friendships, social media connectedness), socio‐behavioral characteristics (social anxiety, disruptive behaviors), and physical (pubertal development) characteristics predicted victimization among young adolescents, using both bully‐reported and self‐reported measures of victimization. Results indicated that the more adolescents deviated from their classroom's norm at the start of the school year, the more likely they were to be increasingly victimized throughout the school year. We observed this pattern among adolescents who had fewer friendships and social media connections (bully‐reported victimization), were more socially anxious (self‐reported victimization) or acted less or more disruptively (both bully‐ and self‐reported victimization) than the norm. Greater deviation from the norm predicted more victimization over time among these individuals.

Moreover, variation around the norm played a role. For disruptive behaviors, short‐term effects of person‐group dissimilarity on bully‐reported victimization were larger when classmates were more *homogeneous* in their disruptive behaviors. By contrast, greater norm‐deviation in terms of social anxiety especially predicted victimization in more *heterogeneous* classrooms, so where classmates were diverse in their levels of anxiety. The similarity in terms of pubertal development did not affect victimization. Multiple sensitivity analyses revealed that the effects of person‐group dissimilarity in relational characteristics and disruptive behaviors on victimization were largely consistent regardless of the exclusion of covariates, outlier corrections, gender group, unilateral or reciprocal friendships measure, type of analytical approach, or method to compute person‐group similarity.

Altogether, the findings indicate that: (1) Not only individual characteristics, but also being *more different* from classroom norms in terms of relational and socio‐behavioral characteristics can predict victimization among young adolescents, (2) When studying person‐group dissimilarity, not only absolute levels of group norms, but also *variation* around the group norm plays a role—for which comprehensive GAPIM formulas were developed; and (3), The characteristics for which dissimilarity predict victimization depend on the reporter, with relational characteristics predicting peer‐nominated victimization and internal processes such as social anxiety predicting self‐reported victimization.

### Deviation from relational and socio‐behavioral group norms as predictors of victimization

Generally, our results provide quantitative evidence for previous suggestions and qualitative findings (e.g., Thörnberg & Delby, 2019) that being different from the norm predicts not only peer rejection (e.g., Boor‐Klip et al., 2017), but also victimization, which is a more extreme consequence of being “different.” It seems especially risky to deviate from relational and socio‐behavioral (i.e., disruptive behavior) norms. Young adolescents’ absolute number of friends was already shown to be associated with victimization (Boulton et al., 1999) but this study adds that the *relative* number of friendships is of importance over and above that absolute number. Having low social resources in terms of friends seems, therefore, less problematic when others also have few friends. Furthermore, dissimilarity in externalizing behaviors has already been associated with social standing (Wright et al., 1986) or victimization (Bass et al., 2021; Brendgen et al., 2015) among children, but with the current longitudinal data, we demonstrate that the associations could last throughout the school year among young adolescents.

Overall, it appears that deviating from norms for *interpersonal* characteristics—those that play a key role in social interactions—predicted victimization, rather than deviations from norms in terms of non‐interpersonal, physical characteristics. Although we can only speculate about the reasons why, it seems possible from a developmental perspective that similarity in interpersonal characteristics is especially important to early adolescent groups because it facilitates reaching developmental goals such as creating intimate peer relationships, collaborating in groups, and forming social identities, all of which are of heightened importance in this age period (Kindermann & Gest, 2018; Laursen & Veenstra, 2021). Adolescents might actively harass those who pose a threat to this homogeneity and fail to “blend in.” In addition, bullies who take lead might benefit from bullying peers who deviate from the group norms. These victims have fewer social resources because they are less defended by bystanders who are dissimilar to them (e.g., Veenstra et al., 2010). Therefore, bullies can signal their power to the group without losing affection.

Noninterpersonal, physical characteristics such as pubertal development might contribute less directly to these social processes. Those who deviate from the norm in their pubertal development might still *behave* in accordance with the norm—for example, regarding dating—to fit in, and thus can form successful peer relationships and build sufficient social resources. Notably, the absence of a significant effect does not prove that there is no effect, so future research should also examine pubertal development, or other, more visible, physical characteristics such as body composition (body mass index).

When focusing on the effects across points in time, results for disruptive behaviors differed throughout the school year. Dissimilarity among those who acted *less* disruptively than the group norm, especially when others were homogeneous, predicted victimization 3 months after the start of the school year. By contrast, greater dissimilarity was also a risk factor for victimization among those who acted *more* disruptively than the group norm, but this effect took 6 months to unfold. This different timing makes sense: In a partly new classroom in which classmates are still exploring their own social position, it might seem dangerous to bully disrespectful individuals who start fights and safer to target those who are less cheeky. However, over time, classmates might dare to punish these disruptive actions in classrooms with a more prosocial norm.

### Cross‐informant findings

Several interesting differences emerged when comparing these main findings that were based on a bully‐report measure of victimization to findings from the sensitivity analyses that were based on a self‐report measure of victimization. Although the self‐report measure produced similar results regarding the short‐term effects of disruptive behaviors, it did not replicate the dissimilarity effects of friendships. Instead, this measure identified additional and consistent effects of dissimilarity in terms of greater social anxiety both in the short and long term, and of a *reciprocal* measure of friendships. Interestingly, these differences between informants align with previous research on the types of victims who are identified with different measures. Victims who are identified through peer‐nomination measures generally are individuals with a poor position in the group, while self‐identified victims are likely to be individuals with negative self‐perceptions (Volk et al., 2017). Indeed, our peer nomination measure identified victims who were dissimilar to others in terms of interpersonal characteristics that were also reported by peers (i.e., received friendship nominations, disruptive behaviors). Thus, bullies who report on their negative behaviors may explicitly do so when their victims are also recognized by others as being in a weaker social position. By contrast, self‐identified victims were especially more socially anxious than the group norm. This social anxiety might be a proxy for insecurity, making adolescents more prone to interpret certain ambiguous situations as “hostile.” Moreover, being socially anxious was self‐reported and reflects an internal experience of affect that may not be visible to peers.

Moreover, only having more reciprocated friendships (and not unilateral, received nominations for) than most peers offers protection against self‐reported victimization: Those who perceive that they have more social support might also perceive themselves less easily to be a victim of bullying. Both measures demonstrated support for person‐group dissimilarity regarding disruptive behavior, which makes sense because this is both an interpersonal construct and a psychological characteristic.

### Strengths and limitations

Strengths of this study include our theory‐driven approach, longitudinal design, large sample, highly advanced analytical approach, and extensive sensitivity analyses. However, this research also has its limitations. First, our measures of victimization were based on peer nominations and could, therefore, reflect only the number of bullies and not the frequency or intensity of victimization. In future studies, self‐report measures that also consider this frequency and severity could be important as well, because the frequency of victimization experiences relates to its impact on well‐being (Solberg & Olweus, 2003). Furthermore, the exclusive use of bully‐reports or victim‐reports of victimization might lead to underestimations of this behavior due to social desirability. Nevertheless, information reported by the broader peer group might have the same effect because of the hidden nature of victimization (Volk et al., 2017). For a comprehensive assessment of victimization, it could be useful to combine self‐reports of victims and bullies with a measure in which classmates report on who is being bullied.

Second, no clear gender moderation effects emerged, but perhaps different patterns would be observed when analyzing gender‐specific norms. Adolescents particularly identify with their same‐gender peers and use them as references for norms (Mehta & Strough, 2010). Moreover, girls typically set the tone in a classroom, when it considers shaping normative beliefs about aggression and influencing externalizing behaviors of both boys and girls (Busching & Krahé, 2015). In the present study, we analyzed gender‐specific norms for pubertal development to understand a phenomenon that is inherently gender‐specific, but doing so for all other characteristics went beyond the already large set of analyses in this study.

Third, at the time of our data collection, most students were already using social media, but today its importance is even greater, and connectedness to others is more visible to others (e.g., through “stories” or likes that are displayed publicly in news updates). As such, it is possible that more recent data would detect larger effects regarding social media connectedness.

Fourth, our predictors were not all assessed at the same time points. While most predictors were assessed in October, social anxiety was assessed in September and social media connectedness in December. Additionally, it is important to study long‐term effects of deviating from the norm regarding social media connectedness.

Finally, caution should be taken in generalizing the results of this study to other countries. For example, adolescents in the Netherlands are relatively low regarding externalizing behaviors compared to young people in other European countries (Hendriks et al., 2020) and the United States (Weeland et al., 2018). Therefore, replicating our analyses in other countries might result in larger effects due to greater variation in the data and potential heterogeneity in norms.

### Implications and future directions

Although most research focuses on individual predictors of victimization, the results of this study reveal that risk factors for victimization should also be examined while considering the group context. Moreover, we demonstrated that the GAPIM approach can be suitable to study person‐group dissimilarity, because it considers variation around the norm, excludes the individual from the norm calculation, and can now also be used to test continuous predictors.

Future studies could build on our findings by examining the pathways through which person‐group dissimilarity may predict victimization. For example, such studies could elucidate whether having fewer defenders can account for the higher risk of victimization among those who deviate from developmentally salient norms (Laursen & Veenstra, 2021). Additionally, studies could test the characteristics for which deviation would predict victimization, for example, whether it concerns especially relational characteristics. Finally, future research could also include measures that consider the frequency of victimization and that consider gender‐specific norms.

A practical implication is that strategies to tackle bullying should not only address individual risks, such as providing social skills training for vulnerable adolescents, but direct attention to the classroom context as well. The finding that students with relatively few friends were at greater risk for being victimized highlights the need for awareness of the troublesome, double‐risk situation of students who have relatively few friends: These adolescents may feel lonely not only because they have fewer friends than their classmates (Lodder et al., 2015), but because they are also at a higher risk of being victimized. Teachers are inclined to place friends together in a classroom, and attention should be paid to individuals who start in a classroom in which they know fewer peers than their classmates. They should receive sufficient opportunities to form new bonds, for example, during activities in subgroups that are not formed based on friendship. Furthermore, one proposed mechanism was that those who are dissimilar to the classroom norms and, thus, part of the “out‐group” are defended less frequently. Inducing empathy can overpower youths’ tendency to help outgroup members less often (Sierksma et al., 2015). Relatedly, universal anti‐bullying programs often include lessons that teach children that individual differences are valuable (Kärnä et al., 2011). Such components might be especially effective to tackle victimization based on norm deviation in adolescent groups.

## CONCLUSIONS

This study shows the important role of deviation from group norms in relation to greater victimization. Dissimilarity in terms of having fewer friendships offline or online and acting less or more disruptively than the classroom norm can be associated with increases in victimization among young adolescents. Future studies and interventions could, thus, look beyond the individual, by considering individuals’ embeddedness in the particular peer group to predict whether they might be at risk for victimization. Researchers could test the pathways through which norm deviation is associated with victimization, such as having fewer defenders, throughout various stages of development.

## CONFLICT OF INTEREST

The authors declare that they have no conflict of interest.

## AUTHOR CONTRIBUTIONS

T.M.L.K. conceived the study, participated in its design and coordination, submitted the preregistration, performed statistical analyses, and drafted the manuscript; L.L.‐W and G.M.A.L. participated in the study design and preregistration, provided support with the statistical analyses and critically revised multiple versions of the draft of the manuscript. All authors read and approved the final manuscript.

## Supporting information

Supplementary Material

## Data Availability

Data are available upon reasonable request and with the permission of the data manager.
