# Supplementary material for: Are victims of bullying primarily social outcasts? Person‐group dissimilarities in relational, socio‐behavioral, and physical characteristics as predictors of victimization
Source: Child Dev. 2022 Apr 20;93(5):1458–74. doi: 10.1111/cdev.13772 (PMC9546482; doi:10.1111/cdev.13772)
Supplement: Supplementary file 1 — Supplementary Material [file CDEV-93-1458-s001.docx]

Supplementary Information

This supplementary information includes detailed information on the estimation procedures (SI 1), analyses on disruptive behaviors for all items separately (SI 2), and analyses that excluded the similarity terms (SI 3). Further, the results of eight sensitivity checks are included:

- SI 4: Analyses on self-reported victimization measure
- SI 5: Analyses without covariates
- SI 6: Analyses without correcting for outliers
- SI 7: Results of gender moderation analyses
- SI 8: Analyses of traditional multilevel models
- SI 9: Analyses with reciprocal measure of friendship
- SI 10: Directional analyses for all measures
- SI 11: Analyses using a product instead of absolute difference term for dissimilarity

SI 1. Details on estimation procedures

We examined outliers on the second (classroom) level based on DFBeta’s, those with a score of > 1 will receive the highest score within the <1 boundary. On the first (individual) level, we replaced outliers that were more than 2.5 *SD* above or below the group mean by the closest possible score within the 2.5 *SD* boundary. We also tested whether the findings differed without correction of outliers (see SI 3).

Regarding missing values, we used the approach of excluding classrooms with fewer <10 students and classrooms with a participation rate < 60% (Garandeau et al., 2021). This was not the case in any classroom: a maximum of 5 individuals was missing in a classroom (for pubertal development, in the girls sample).

We used a mixed (individuals nested in classrooms) Poisson regression approach because our outcome was a skewed count variable with many zero’s (85%). Overall, the Poisson distribution showed large overlap with the observed data distribution (see Figure S1) and means and variances were close (difference between means and variances = .04 on T2 on a 0-2 scale and .05 on T3 on a 0-4 scale), supporting the decision for the Poisson regression approach. The regression was performed separately for short-term effects on victimization (T2, December) and long-term effects (T3, March).

Our FDR-controlling procedure included that we first ordered *p* values across the six (Table 3) from smallest to largest, ranking them *i* = 1 to *i* = 5. A threshold of significance (critical value) was established according to the formula: critical value value (*p_i_*) = $\frac{i}{m}$ *Q* (*m* = number of tests, *Q* = percentage of false discoveries 5% = .05). This procedure resulted in the following critical values: *p*(1) ≤ .002, *p*(2)≤ .02, *p*(3) ≤ .03, *p*(4) ≤ .03, *p*(5) ≤ .04, *p*(6) ≤ .05. Each ranked *p* value was then compared with its corresponding critical value: that of the highest ranking *p* value that was below its corresponding critical value. Thus, the lowest *p* value was compared to p*(*1) ≤ .002, the second lowest to *p*(2) ≤ .02, and so on.

**Recoding of variables**

Independent variables used in (except for covariates) were coded to range from -1 to 1 using the formula described by Garcia and colleagues (2015): 2(X_ik_ − X_S_)/(X_L_ − X_S_) – 1, where X is the individual predictor score from person *i* in classroom *k*, where X_S_ is the smallest observed value in the classroom and where X_L_ is the largest observed value. As a result, the similarity terms *i* and *i’* may equal 1 when there is maximal similarity and -1 when there is maximal dissimilarity.

Figure S1. *Fit of Observed Versus Poisson Distribution of Victimization at T2 and T3.*

SI 2. Results for disruptive behavior: All three items separately

Analyses for all items of the disruptive behaviors measure separately showed similar results as the main analyses with the composite measure, although some effects showed trend effects instead of significant effects (Tables S8, S9). The item about being cheeky to teachers showed a marginally significant person-group similarity effect for the short-term person-fit model (*IRR =* 0.47), but a contrast interaction effect that showed that being *less* cheeky than the norm predicted more victimization in homogeneous classrooms (*b*_i_ *=* -1.67, 95% CI = -2.63; -0.70, *IRR =* 0.19) and not in more heterogeneous classrooms (*b_i_* = -1.52, 95% CI = -4.55; 1.52, *IRR =* 0.22). For being more cheeky than the norm, effects were non-significant both in more homogeneous (*b_i_* =-1.08, 95% CI = -6.91; 4.75, *IRR =* 0.34) and heterogeneous (*b*_i_ = -0.02, 95% CI = -2.24; 2.19, *IRR =* 0.98) classrooms. In the long term, the person-fit model fitted the data best (*IRR =* 0.38) and showed that being more dissimilar to the peer group-norm predicted more victimization. Among those who were *less* cheeky than the norm, similarity did not predict victimization (*b*_i_ *=* 0.00, 95% CI = -0.77; 0.77, *IRR =* 0.00) while among those who were *more* cheeky, more dissimilarity predicted more victimization (*b_i_* = -2.30, 95% CI = -3.94; -0.66, *IRR =* 0.10).

For fighting, the person-fit model fitted the data best in the short term and long term but showed no significant person-group similarity ffect in the short-term (*IRR =* 0.69) and only a marginally significant negative effect in the long-term (*p = .*06; *IRR* = 0.57), in the direction of dissimilarity.

For breaking rules, the short-term effect showed no significant similarity effects in any of the models (*IRR* = 0.82), but the long-term effect model did (*IRR* = 0.32): a dissimilarity effect on victimization was shown among those who showed *more* rule-breaking behavior than the norm (*b*_i_ *= -*1.28, 95% *CI* = -2.48; -0.07; *IRR =* 0.28) and not among those who showed *less* rule-breaking behavior (*b_i_ =* -0.55, 95% *CI* = -1.32; 0.21; *IRR =* 0.58).

Table S1. *For All Disruptive Behavior Items Separately: Comparison of Poisson GAPIM Submodels For Individual-Group (Dis)Similarity Effects on Bully-Reported Victimization.*

|  | T2 | | | | | |  | T3 | | | | | |
| --- | --- | --- | --- | --- | --- | --- | --- | --- | --- | --- | --- | --- | --- |
| Characteristics at baseline (T1: October) | Person-fit | | Complete | | Contrast interaction | |  | Person-fit | | Complete | | Contrast interaction | |
|  | *AIC* | *BIC* | *AIC* | *BIC* | *AIC* | *BIC* |  | *AIC* | *BIC* | *AIC* | *BIC* | *AIC* | *BIC* |
| Cheeky | 1244.2 | 1300.7 | 1245.6 | 1307.3 | **1235.5** | **1302.4** |  | **1183.1** | **1239.6** | 1185.2 | 1246.7 | 1186.4 | 1253.2 |
| Fights | **1240.1** | **1296.6** | 1241.0 | 1302.8 | 1239.7 | 1306.5 |  | **1182.9** | **1239.4** | 1184.71 | 1246.4 | 1185.8 | 1252.6 |
| Rule-breaking | **1248.2** | **1304.8** | 1249.0 | 1310.7 | 1249.9 | 1316.8 |  | **1178.5** | **1235.0** | 1180.1 | 1241.8 | 1180.3 | 1247.1 |

AIC = Akaike Information Criterion. BIC = Bayesian Information Criterion.

Table S2. *GAPIM Submodels: Poisson Estimations of Individual-Group (Dis)Similarity Effects of Separate Items of Disruptive behaviors on Victimization*.

|  | T2 |  | T3 |  |
| --- | --- | --- | --- | --- |
| Characteristics at baseline (T1: October) | *b* | 95% *CI* | *b* | 95% *CI* |
| Cheeky |  |  |  |  |
| *Person-fit model* |  |  |  |  |
| Person score *x* | -0.34 | -0.88; 0.19 | **-0.49** | -0.97; -0.01 |
| Group score *x’* | 0.36 | -1.04; 1.76 | -1.11 | -2.61; 0.38 |
| Person-group similarity *i* | -0.75^1^ | -1.52; 0.03 | **-0.98** | -1.66; -0.29 |
| Contrast interaction model |  |  |  |  |
| Person score x | 0.18 | -0.47; 0.82 |  |  |
| Group score x’ | 0.88 | -1.46; 3.22 |  |  |
| Person-group similarity i | 1.17 | -0.19; 2.53 |  |  |
| Group-group similarity i’ | 0.75 | -1.38; 2.88 |  |  |
| Contrast interaction (*i*i’*) | **-2.75** | -4.29; -1.22 |  |  |
| Fights |  |  |  |  |
| Person score *x* | -0.38 | -1.51; 0.75 | -0.03 | -1.08; 0.95 |
| Group score *x’* | -0.71 | -3.47; 2.06 | 1.25 | -1.49; 3.99 |
| Person-group similarity *i* | -0.90 | -1.98; 0.18 | -0.59^2^ | -1.57; 0.38 |
| Rule-breaking |  |  |  |  |
| Person score *x* | -0.06 | -0.69; 0.57 | **-0.52** | -1.05; -0.00 |
| Group score *x’* | 0.33 | -1.02; 1.67 | -0.57 | -2.01; 0.86 |
| Person-group similarity *i* | -0.20 | -0.95; 0.55 | **-1.14** | -1.81; -0.46 |

*Note.* Numbers in bold represent significant findings. ^1^*p* = .07. ^2^ *p* *= .*06.

SI 3: Person and group effects

Models that excluded the similarity effects were estimated to observe main effects of individual and descriptive classroom norm levels of the characteristics (Table S3). For relational characteristics, results showed for both short term and long term effects, that individuals who had *fewer* friends and who were in classrooms with a descriptive norm for having more friends or having *more* social media connections than in “average” (grand mean) classrooms were more likely to be victimized later in the school year. Regarding socio-behavioral characteristics, only having higher levels of disruptive behaviors predicted long-term victimization, and there were no effects of anxiety on victimization on either the individual or classroom level. Last, regarding pubertal development, boys who reported *less* advanced pubertal development were more likely to be victimized in the long term, and girls who reported *more* advanced pubertal development and who were in classrooms with a descriptive norm for *more* advanced pubertal development were also more likely to be victimized in the short term.

Table S3. *Preliminary Models: Poisson Estimations of Individual and Group Norm Effects on Bully-Reported Victimization*.

|  | T2 |  | T3 |  |
| --- | --- | --- | --- | --- |
| Characteristics at baseline (T1: October) | *b* | 95% *CI* | *b* | 95% *CI* |
| Friendships |  |  |  |  |
| Person score *x* | **-1.33** | -1.69; -0.98 | **-1.11** | -1.46; -0.75 |
| Group score *x’* | **1.47** | 0.35; 2.58 | **1.24** | 0.06; 2.43 |
| Social media connectedness |  |  |  |  |
| Person score *x* | *n/a* | *n/a* | -0.13 | -0.32; 0.06 |
| Group score *x’* | *n/a* | *n/a* | **1.96** | 0.70; 3.22 |
| Social anxiety |  |  |  |  |
| Person score *x* | 0.08 | -0.16; 0.32 | 0.09 | -0.17; 0.34 |
| Group score *x’* | -0.52 | -2.50; 1.46 | -0.79 | -2.82; 1.24 |
| Disruptive behavior |  |  |  |  |
| Person score *x* | 0.18 | -0.06; 0.41 | **0.24** | 0.01; 0.47 |
| Group score *x’* | 0.74 | -0.83; 2.31 | -0.29 | -1.96; 1.38 |
| Pubertal development |  |  |  |  |
| Boys |  |  |  |  |
| Person score *x* | -0.07 | -0.59; 0.45 | **-0.72** | -1.31; -0.14 |
| Group score *x’* | 2.03 | -0.42; 4.47 | 1.33 | -1.29; 3.94 |
| Girls |  |  |  |  |
| Person score *x* | **0.67** | 0.08; 1.25 | -0.35 | -0.91; 0.21 |
| Group score *x’* | **2.04** | 0.60; 5.48 | -0.75 | -3.17; 1.67 |

*Note.* Numbers in bold represent significant findings.

SI 4. Results of analyses with a self-reported measure of victimization

Table S4 shows the Pearson intercorrelations with the self-reported victimization variable and Table S5 shows the model fit (AIC/BIC) of the different GAPIM submodels for every characteristic on self-reported victimization. Table 4 (manuscript) shows the results for the best-fitting model. Overall, AIC and BIC indices were larger than those in the main analyses, indicating that these analyses fitted worse than the analyses using the bully-reported measure.

**Relational Characteristics**

For all models of relational characteristics (friendships in short- and long-term and social media connections), the person-fit model showed the best fit and the other models did not show additional significant effects. No person-group similarity effects were observed, so in contrast to results for bully-reported victimization, having fewer friends or fewer social media connections did not predict self-reported victimization.

**Socio-Behavioral Characteristics**

For anxiety, for both the short- and the long-term effects the contrast interaction model fitted best and showed significant contrast interaction effects. The person-fit model did not differ substantially in terms of model fit (Δ AIC/BIC<10), so we reported both models for each time point, starting with the most parsimonious person-fit models. The complete models did not show a significant group similarity effect so these were not reported. For the short-term effects, a significant negative person-group similarity effect was observed (*IRR =* 0.04). A higher dissimilarity between individuals’ levels of anxiety and the norm predicted more self-reported victimization, among those higher in anxiety than the norm. Directional follow-up analyses showed that this effect was greatest in more *heterogeneous* classrooms (*b*_i_ = -3.82, 95% *CI* = -5.40; -2.23, *IRR =* 0.01) and absent in more *homogeneous* classrooms (*b*_i_ = 1.52, 95% *CI* = -4.49; 7.54, *IRR =* 53.9). Given the *IRR,* with every *SD* (= 0.29) decrease in similarity, one’s number of bullies in heterogeneous classrooms demonstrated a 29% increase in the short term. For long-term effects, a significant negative person-group similarity effect was observed again, so a higher discrepancy between individuals’ higher levels of anxiety than the norm predicted more self-reported victimization in the long-term too (*IRR =* 0.01). Again, directional follow-up analyses showed that this effect was greater in more *heterogeneous* classrooms (*b*_i_ = -3.37, 95% *CI* = -5.83; -0.91, *IRR =* 0.03) than in more *homogeneous* classrooms (*b*_i_ = 3.15, 95% *CI* = -0.35; 6.65, *IRR =* 23.4). Given the *IRR,* with every *SD* decrease in similarity, one’s number of bullies in heterogeneous classrooms demonstrated a 28% increase in the long term. Thus, person-group dissimilarity effects were in the expected direction, but effects of homogeneity were in the reverse direction. When individuals differed more from the peer-group norm in terms of being more anxious, they were victimized more – especially when in that classroom their peers differed in the extent to which they were anxious (e.g., some peers were much more anxious than others).

For disruptive behavior, regarding short-term effects on victimization, the person-fit model fitted the data best and the other models also did not show significant additional effects. The model showed a negative person-group similarity effect (*IRR =* 0.14): being more dissimilar to the peer-group norm in disruptive behaviors predicted more victimization. In line with the bully-reported victimization analyses, follow-up analyses showed that more dissimilarity among those who acted *less* disruptively than the norm predicted more victimization (*b_i_ =* -1.93, 95% *CI* = -2.69, -1.17; *IRR =* 0.14). For those who acted *more* disruptively than the norm, the person-group similarity effect on victimization was again not significant (*b_i_ =* 0.44, 95% *CI* = -0.91, 1.79; *IRR =* 1.55). Given the *IRR,* with every *SD* (= 0.21) decrease in similarity, one’s number of bullies in heterogeneous classrooms increased with 24% among those who acted *less* disruptively than the norm.

Further, for the long-term effects of self-reported victimization, the person-fit model fitted best but did not show significant effects, and neither did the other models. Thus, (only) the short-term effects replicated the results of the main analyses in showing that more dissimilarity from others in terms of acting less disruptively predicted more victimization.

**Physical Characteristics**

For pubertal development, for boys, in the short term the complete model fitted best but showed no person-group similarity effect. In the long term, the person-fit model did not show person-group similarity effects, but the contrast interaction model showed a significant contrast interaction effect. However, in the directional follow-up analyses, none of the analyses showed a significant effect of person-group similarity.

For girls, both for the short- and long-term effects models, the person-fit models fitted the data best but showed no significant person-group similarity effects, and the other models also showed no additional significant effects, like the main analyses. Deviating from same-sex classmates in one’s stage of pubertal development thus did not significantly predict self-reported victimization for girls.

**Conclusion** In sum, results complemented those found in the analyses using the peer-nomination measure of victimization, such that dissimilarity in relational characteristics did not predict victimization, but dissimilarity in terms of socio-behavioral characteristics did. Higher dissimilarity to the classroom norm among those who were more socially anxious than the norm predicted more victimization –especially in heterogeneous classrooms with a few highly anxious peers, both after three and six months. For disruptive behavior in the short term (three months) the effects were similar to the main model based on bully-reports of victimization, except that homogeneity did not affect the results.

Table S4. *Pairwise Pearson Correlations of Self-Reported Victimization with All Variables.*

| Variables | 1. | 2. | 3. |
| --- | --- | --- | --- |
| 1. Victimization (self-reported) T1 | -- |  |  |
| 2. Victimization (self-reported) T2 | .52** | -- |  |
| 3. Victimization (self-reported) T3 | .27** | .35** | -- |
| 4. Victimization (bully-reported) T1 | .32** | .25** | .17** |
| 5. Victimization (bully-reported) T2 | .22** | .33** | .26** |
| 6. Victimization (bully-reported) T3 | .17** | .14** | .17** |
| 7. Social anxiety T1 | .11** | .08** | .02 |
| 8. Disruptive behaviors T1 | .08** | .09* | .05 |
| 9. Friendships T1 | -.14** | -.14** | -.08* |
| 10. Social media connectedness T2^1^ | -.06* | -.06* | -.04 |
| 11. Boys’ pubertal development T1 | -.01 | -.01 | -.01 |
| 12. Girls’ pubertal development T1 | .04 | .04 | -.03 |

^1^Social media connectedness was only measured at T2. **p* < .05 ** *p <* .001. Self-reported victimization means are for T1 *M*= 0.29, *SD* = 1.20, for T2 *M*= 0.40, *SD* = 1.74; for T3 *M*= 0.34, *SD* = 1.74.

Table S5. *Comparison of Poisson GAPIM Submodels For Individual-Group (Dis)Similarity Effects on Self-Reported Victimization*

|  | T2 | | | | | |  | T3 | | | | | |
| --- | --- | --- | --- | --- | --- | --- | --- | --- | --- | --- | --- | --- | --- |
| Characteristics at baseline (T1: October) | Person-fit | | Complete | | Contrast interaction | |  | Person-fit | | Complete | | Contrast interaction | |
|  | *AIC* | *BIC* | *AIC* | *BIC* | *AIC* | *BIC* |  | *AIC* | *BIC* | *AIC* | *BIC* | *AIC* | *BIC* |
| Friendships | **1074.2** | **1117.8** | 1076.0 | 1123.9 | 1076.9 | 1129.2 |  | **1173.2** | **1216.5** | 1174.9 | 1222.5 | 1176.1 | 1228.1 |
| Social media connectedness |  |  |  |  |  |  |  | **1066.8** | **1110.6** | 1068.8 | 1116.4 | 1069.7 | 1122.3 |
| Social anxiety | **931.2** | **973.6** | 930.7 | 977.2 | **920.8** | **971.5** |  | **867.4** | **909.7** | 868.7 | 915.2 | **859.8** | **910.6** |
| Disruptive behavior | **2014.6** | **2070.8** | 2014.6 | 2075.9 | 2014.6 | 2080.9 |  | **2166.5** | **2222.5** | 2166.5 | 2227.6 | 2167.0 | 2233.2 |
| Boys’ pubertal development | 1067.1 | 1110.8 | **1060.9** | **1109.0** | 1061.1 | 1113.6 |  | 1153.0 | 1196.7 | 1149.8 | 1202.2 | **1152.3** | **1200.3** |
| Girls’ pubertal development | **856.6** | **900.4** | 858.4 | 906.8 | 858.4 | 911.3 |  | **886.4** | **930.4** | 888.1 | 940.7 | 886.6 | 935.0 |

AIC = Akaike Information Criterion. BIC = Bayesian Information Criterion.

SI 4. Sensitivity check: Results of analyses without covariates

Regarding friendships, for the short- and long-term effects, the person-fit model fitted the data best and the other models did not show significant group-group similarity effects or a contrast interaction effect. The person-fit models showed significant negative person-group similarity effects (for T2, *IRR =* 0.21; for T3, *IRR =* 0.16). This means that being more dissimilar from the peer-group norm in terms of having fewer friendships predicted more victimization on the short and long term. The only difference with the main analyses was that variation in the homogeneity of others to each other did not affect the outcomes.

For social media connectedness, the person-fit model fitted the data best and the other models did not show significant group-group similarity effects or a contrast interaction effect. The person-fit model showed, similar to the main analyses, a significant negative person-group similarity effect *(IRR* = 0.12): Being more dissimilar to the peer-group norm in terms of having fewer social media connections predicted more victimization.

For anxiety, for both the short- and the long-term effects the person-fit models fitted the data best, but showed no significant effects (*IRR =* 2.51 for T2, *IRR* = 1.98 for T3). Similar to the main analyses, being dissimilar from the norm in terms of being less anxious did not significantly predict victimization.

For disruptive behaviors, regarding the short-term effect, similar to the main analyses the complete model fitted the data best (but not the contrast interaction model, as was the case in the main analyses) and included significant group-group similarity effects. The complete model showed a significant negative person-group similarity effect (*IRR =* 0.16). Being more dissimilar in individual disruptive behaviors to the peer group-norm predicted more victimization, while considering the variation in the extent to which other peers were homogeneous to each other in terms of disruptive behaviors – with more homogeneity predicting higher levels of victimization. Follow-up analyses showed that being dissimilar in terms of disruptive behaviors predicted victimization both in terms of acting *less* disruptively than the peer-group norm (*b*_i_ = -1.54, 95% *CI* = -2.26; -0.82, *IRR* = 0.14) and in terms of acting *more* disruptively (*b*_i_ = -1.97, 95% *CI* = -0.10; -3.65, *IRR* = 0.21). In the main analyses, the latter effect only showed a trend and no significant effect, and the contrast interaction model showed a similar fit. For the long-term effect, the person-fit model and contrast interaction model fitted the data best. The person-fit model showed a similarity effect (*IRR =* 0.14): Being dissimilar in terms of disruptive behaviors predicted victimization in terms of acting *less* disruptively than the peer-group norm (*b*_i_ = -2.53, 95% *CI* = -3.23; -1.83, *IRR* = 0.08) and not in terms of acting *more* disruptively (*b*_i_ = -1.30, 95% *CI* = -0.3.00; 0.39, *IRR* = 0.27). The contrast interaction model additionally showed that this effect of acting less disruptively was stronger in more heterogeneous (*b*_i_ = -3.37, 95% *CI* = -5.09; -1.66, *IRR* = 0.03) classrooms than in homogeneous classrooms (*b*_i_ = -1.61, 95% *CI* = -2.99; -0.24, *IRR* = 0.20), although this comparison should be interpreted cautiously given the relatively large standard errors in the heterogeneous classrooms sample. Dissimilarity in terms of acting *more* disruptively than the peer-group norm did not predict victimization in either heterogeneous (*b*_i_ = 0.24, 95% *CI* = -2.08; 2.56, *IRR* = 1.27), or homogeneous (*b*_i_ = -2.21, 95% *CI* = -5.59; 1.42, *IRR* = 0.11), classrooms.

For pubertal development, both for boys and girls the short- and long-term effects models, the person-fit models fitted the data best but showed no significant person-group similarity effects, and the other models also showed no additional significant effects. Deviating from same-sex classmates in one’s stage of pubertal development thus did not significantly predict victimization in this sample. Homogeneity of the group norms did also not contribute to the effects. This is similar to the main analyses.

Altogether, results without covariates were overall similar to the main analyses in terms of revealing person-group dissimilarity effects for friendships, social media connectedness, and disruptive behaviors. The role of homogeneity of group norms was less clear than in the main analyses, showing in the long term (instead of short term) both in more heterogeneous and homogeneous classrooms dissimilarity effects. Also similar to the main analyses, no similarity effects were found for social anxiety and pubertal development.

Table S6. *Without Covariates: Comparison of Poisson GAPIM Submodels For Individual-Group (Dis)Similarity Effects on Bully-Reported Victimization*

|  | T2 | | | | | |  | T3 | | | | | |
| --- | --- | --- | --- | --- | --- | --- | --- | --- | --- | --- | --- | --- | --- |
| Characteristics at baseline (T1: October) | Person-fit | | Complete | | Contrast interaction | |  | Person-fit | | Complete | | Contrast interaction | |
|  | *AIC* | *BIC* | *AIC* | *BIC* | *AIC* | *BIC* |  | *AIC* | *BIC* | *AIC* | *BIC* | *AIC* | *BIC* |
| Friendships | **839.5** | **857.1** | 841.5 | 863.5 | 841.6 | 868.0 |  | 802.1 | 819.7 | **797.4** | **819.4** | 798.2 | 824.6 |
| Social media connectedness |  |  |  |  |  |  |  | **702.1** | **719.8** | 704.0 | 726.1 | 705.4 | 731.9 |
| Social anxiety | **559.3** | **576.3** | 560.4 | 581.7 | 561.7 | 587.3 |  | **499.9** | **516.9** | 501.9 | 523.1 | 503.8 | 529.3 |
| Disruptive behavior | **1359.5** | **1385.2** | 1355.7 | 1386.6 | 1343.6 | 1379.6 |  | **1279.8** | **1305.5** | 1281.6 | 1312.5 | 1283.5 | 1319.2 |
| Boys’ pubertal development | **751.0** | **773.1** | **751.3** | **777.8** | 753.3 | 784.2 |  | **674.7** | **696.8** | 672.2 | 698.7 | 671.7 | 702.6 |
| Girls’ pubertal development | **566.9** | **589.0** | 568.8 | 569.4 | 569.2 | 600.2 |  | **588.0** | **610.1** | 586.6 | 613.2 | 588.6 | 619.6 |

AIC = Akaike Information Criterion. BIC = Bayesian Information Criterion.

Table S7. *Without Covariates:* *Poisson Estimations of Individual-Group (Dis)Similarity Effects on Bully-Reported Victimization*.

|  | T2 |  | T3 |  |
| --- | --- | --- | --- | --- |
|  |  |  |  |  |
| Characteristics at baseline (T1: October) | *b* | 95% *CI* | *b* | 95% *CI* |
| *Directional analyses^1^* | | | | |
| Friendships |  |  |  |  |
| *Person-fit/complete model* |  |  |  |  |
| Person score *x* | **-1.35** | -2.25; -0.44 | -0.33 | -1.29; 0.61 |
| Person-group similarity *i* | **-1.56** | -2.74; -0.37 | **-2.22** | -3.43, -1.01 |
| Group-group similarity *i’* |  |  | 3.58 | 1.10; 6.06 |
| Social media connectedness |  |  |  |  |
| *Person-fit model* |  |  |  |  |
| Person score *x* |  |  | **0.88** | 0.26; 1.49 |
| Person-group similarity *i* |  |  | **-2.15** | -3.26; -1.05 |
| Social anxiety |  |  |  |  |
| *Person-fit model* |  |  |  |  |
| Person score *x* | 1.24 | -0.62; 3.09 | 1.22 | -0.84; 3.27 |
| Person-group similarity *i* | 0.92 | -2.02; 3.86 | 0.68 | -2.61; 3.98 |
| *Non-directional analyses* | | | | |
| Disruptive behavior |  |  |  |  |
| *Person-fit/complete model* |  |  |  |  |
| Person score *x* | **-1.02** | -1.63; -0.41 | **-0.83** | -1.32; -0.33 |
| Group score *x’* | 3.69 | 0.90; 6.48 | 1.03 | -0.34; 2.39 |
| Person-group similarity *i* | **-1.85** | -2.73; -0.97 | **-1.94** | -2.68; -1.20 |
| Group-group similarity *i’* | 3.20 | 0.61; 5.78 |  |  |
| *Contrast interaction model* |  |  |  |  |
| Person score *x* |  |  | -0.01 | -0.55; 0.53 |
| Group score *x’* |  |  | -0.00 | -2.59; 2.58 |
| Person-group similarity *i* |  |  | -0.12 | -1.29; 1.05 |
| Group-group similarity *i’* |  |  |  |  |
| Contrast interaction (*i*i’*) |  |  | **-2.07** | -3.42; -0.71 |
| Pubertal development |  |  |  |  |
| Boys |  |  |  |  |
| *Person-fit/complete model* |  |  |  |  |
| Person score *x* | **-0.24** | **-**0.68; 0.20 | **-0.70** | -1.19; -0.21 |
| Group score *x’* | 1.69 | -0.05; 3.34 | 2.51 | 0.55; 4.46 |
| Person-group similarity *i* | -0.28 | -1.18; 0.62 | -0.53 | -1.59; 0.54 |
| Girls |  |  |  |  |
| *Person-fit model* |  |  |  |  |
| Person score *x* | 0.36 | -0.17; 0.89 | -0.11 | -0.61; 0.40 |
| Group score x*’* | 2.14 | 0.36; 3.93 | 0.89 | -0.96; 2.75 |
| Person-group similarity *i* | -0.19 | -1.25; 0.87 | -0.55 | -1.67; 0.56 |

*Note.* Numbers in bold represent significant findings at the individual level. ^1^Directional analyses were conducted by estimating the effect only among individuals who scored *≤* (friendships, social media connectedness) or *≥* (social anxiety) than the classroom norm *x’*.

SI 6. Sensitivity check: Results of analyses without correction for outliers

No outliers on the second (classroom) level were found, based on DFBeta’s using the >1 cut-off value (highest value = -0.56). However, we corrected for outliers on the first level, defined as scoring lower or higher than 2.5 *SD* from the mean, where outlier percentages were 2% (social anxiety), 4% (disruptive behavior), 1% (friendships) and 0% (social media connectedness, pubertal development).

Analyses (Tables S8, S9) showed that for friendships, in the short term the person-fit model fitted best and showed a dissimilarity effect (*IRR =* 0.24), and in the long term the complete model fitted the data best and showed a dissimilarity effect as well (*IRR* = 0.12), while taking into account variation in the extent to which other peers were homogeneous to each other in terms of friendships – with more homogeneity predicting higher levels of victimization.

For social anxiety, again the person-fit models fitted best but showed no effects. For disruptive behaviors, in the short term, both the person-fit and the contrast interaction model fitted the data best (*IRR =* 0.26). Follow-up analyses showed again that acting less disruptive than the peer-group norm predicted more victimization (*b*_i_ = -1.32, 95% *CI* = -2.62; -0.04, *IRR* = 0.14) while acting more disruptive than the peer norm did not show an effect of dissimilarity on victimization (*b*_i_ = -1.95, 95% *CI* = -4.41; 0.51, *IRR* = 0.27). Further, again, the contrast interaction model additionally showed that more dissimilarity by acting less disruptive than the norm especially predicted victimization in *more* *homogeneous* classrooms (*b* = -2.22, 95% *CI* = -3.57; -0.88, *IRR =* 0.10) and not in heterogeneous classrooms (*b* = 2.28, 95% *CI* = -0.58; 5.14, *IRR =* 42.3). Again, there were no person-group similarity effects of acting more disruptively in either homogeneous (*b* = -0.98, 95% *CI* = -5.23; 3.26, *IRR =* 0.37) or heterogeneous (*b* = 1.63, 95% *CI* = -2.23; 5.50, *IRR =* 5.13) classrooms, but this should be interpreted with caution considering the small number of victimization cases in these contexts (*N* = 21 in heterogeneous sample).

In the long term, again similar to the main analyses, the person-fit model fitted best, and the other models did also not show significant group-group similarity effects or a contrast interaction effect. The model showed a negative person-group similarity effect (*IRR =* 0.13): being more dissimilar to the peer-group norm in disruptive behaviors predicted more victimization. Directional follow-up analyses showed again that being dissimilar in terms of acting *less* disruptive than the classroom norm did not predict victimization (*b* = -0.28, 95% CI = -1.55; 0.99, *IRR* = 0.76) but that acting *more* disruptive did (*b* = -2.53, 95% CI = -4.90; -0.15, *IRR* = 0.08). Altogether, these findings were the same as those found in the main analyses that corrected for outliers.

Table S8. *No Correction for Outliers: Comparison of Poisson GAPIM Submodels For Individual-Group (Dis)Similarity Effects on Bully-Reported Victimization.*

|  | T2 | | | | | |  | T3 | | | | | |
| --- | --- | --- | --- | --- | --- | --- | --- | --- | --- | --- | --- | --- | --- |
| Characteristics at baseline (T1: October) | Person-fit | | Complete | | Contrast interaction | |  | Person-fit | | Complete | | Contrast interaction | |
|  | *AIC* | *BIC* | *AIC* | *BIC* | *AIC* | *BIC* |  | *AIC* | *BIC* | *AIC* | *BIC* | *AIC* | *BIC* |
| Friendships | **787.6** | **831.7** | 789.4 | 837.9 | 789.7 | 842.6 |  | 735.8 | 779.9 | **730.7** | **779.1** | 732.2 | 785.0 |
| Social anxiety | 494.9 | 537.4 | 495.0 | 541.8 | 495.6 | 546.6 |  | **443.8** | **486.3** | 445.8 | 492.5 | 447.2 | 498.1 |
| Disruptive behavior | 1242.6 | 1299.2 | 1240.9 | 1302.6 | 1228.8 | 1295.7 |  | **1175.5** | **1232.0** | 1177.4 | 1239.0 | 1179.4 | 1246.2 |

AIC = Akaike Information Criterion. BIC = Bayesian Information Criterion.

Table S9. *No Correction for Outliers* *GAPIM Submodels: Poisson Estimations of Individual-Group (Dis)Similarity Effects on Self-Reported Victimization*.

|  | T2 | | T3 | |
| --- | --- | --- | --- | --- |
| Characteristics at baseline (T1: October) | *b* | 95% *CI* | *b* | 95% *CI* |
| Friendships |  |  |  |  |
| *Person-fit/complete model* |  |  |  |  |
| Person score *x* | -1.10 | -2.24; 0.03 | **-0.09** | -1.31; 1.13 |
| Person-group similarity *i* | **-1.42** | **-**2.73; -0.10 | **-2.08** | -3.44; -0.71 |
| Group-group similarity *i’* |  |  | **3.43** | -3.44; -0.71 |
| Social anxiety |  |  |  |  |
| *Person-fit model* |  |  |  |  |
| Person score *x* | -0.99 | -4.45; 2.49 | -0.75 | -4.38; 2.87 |
| Person-group similarity *i* | -1.48 | -5.55; 2.59 | -1.66 | -6.02; 2.69 |
| Disruptive behavior |  |  |  |  |
| *Person-fit model* |  |  |  |  |
| Person score *x* | -0.84 | -2.00; 0.32 | **-1.27** | -2.21; -0.32 |
| Group score *x’* | 0.25 | -2.65; 3.15 | -1.85 | -4.81; 1.12 |
| Person-group similarity *i* | **-1.36** | -2.66; -0.07 | **-2.03** | -3.09; -0.96 |
| *Contrast interaction model* |  |  |  |  |
| Person score x | 0.29 | -1.08; 1.66 |  |  |
| Group score *x’* | 4.21 | -1.09; 9.50 |  |  |
| Person-group similarity *i* | 3.15 | 0.46; 5.84 |  |  |
| Group-group similarity *i’* | **4.49** | 0.87; 8.10 |  |  |
| Contrast interaction (*i*i’*) | **-5.90** | -8.85; -2.85 |  |  |

*Note.* Numbers in bold represent significant findings.

SI 7: Gender moderation

We performed additional exploratory analyses to examine whether the similarity effects were different across gender groups. We added interactions with the dummy variable sex (*0* = girl, *1* = boy) to the person score, the group score (in the non-directional analyses) and the similarity score. To test whether homogeneity of norms contributed to the results, we also tested contrast interaction models by adding the *gender*i*i’* interaction effect (see Tables S10 and S11).

For friendships, social media connections, and anxiety, no significant gender moderation effects were found. For disruptive behaviors, a significant gender moderation effect was found for T3 only. Gender-stratified results showed for girls a significant negative person-group similarity effect (*IRR =* 0.16). However, follow-up results showed no similarity effect for girls who acted either less disruptive than the norm (*b*_i_ *=* -0.26, 95% *CI* = -1.59; 1.09, *IRR =* 0.77) or who acted more disruptive than the norm (*b*_i_ *=* -0.94, 95% *CI* = -3.40; 1.51, *IRR =* 0.40). However, the gender-stratified results showed for boys a significant negative person-group similarity effect (*IRR =* 0.34). Follow-up results showed no similarity effect for boys who acted *less* disruptive than the norm (*b*_i_ *=* -0.32, 95% *CI* = -1.58; 0.94, *IRR =* 0.72), but a significant similarity effect was shown for boys who acted *more* disruptive than the norm (*b*_i_ *=* -3.01, 95% *CI* = -5.66; -0.36, *IRR =* 0.05).

In conclusion, these sensitivity analyses showed that most results were not moderated by gender, except that for long-term effects of disruptive behaviors, the effect of acting more disruptive than the norm seemed stronger for boys than for girls. However, it should be noted that this conclusion is based on the non-significant directional follow-up analyses for girls, yet a significant omnibus person-group dissimilarity effect was found for girls as well.

Table S10. *Gender Moderation: Comparison of Poisson GAPIM Submodels For Individual-Group (Dis)Similarity Effects on Bully-Reported Victimization with Gender Interactions.*

|  | T2 | | | | | |  | T3 | | | | | |
| --- | --- | --- | --- | --- | --- | --- | --- | --- | --- | --- | --- | --- | --- |
| Characteristics at baseline (T1: October) | Person-fit | | Complete | | Contrast interaction | |  | Person-fit | | Complete | | Contrast interaction | |
|  | *AIC* | *BIC* | *AIC* | *BIC* | *AIC* | *BIC* |  | *AIC* | *BIC* | *AIC* | *BIC* | *AIC* | *BIC* |
| Friendships | **782.6** | **835.3** | 812.2 | 865.1 | 811.8 | 869.0 |  | 811.8 | 869.0 | **735.0** | **792.2** | 740.2 | 801.7 |
| Social media connections | *n/a* | *n/a* | *n/a* | *n/a* | *n/a* | *n/a* |  | **619.9** | **672.9** | 621.4 | 678.8 | 626.3 | 696.9 |
| Social anxiety | **495.9** | **547.0** | 494.9 | 550.3 | 489.6 | 557.7 |  | **449.3** | **500.3** | 451.2 | 506.4 | 454.9 | 522.9 |
| Disruptive behavior | **1240.1** | **1312.1** | 1238.4 | 1315.6 | 1229.3 | 1311.6 |  | **1168.2** | **1240.2** | 1170.2 | 1247.2 | 1180.8 | 1262.9 |

AIC = Akaike Information Criterion. BIC = Bayesian Information Criterion. Numbers in bold refer to the final model(s), which were multiple models if Δ BIC<10 and an additional effect (of *i'* in the complete model, or of *i*i'* in the contrast interaction model) was observed.

Table S11. *Gender Moderation: Poisson Estimations of Individual-Group (Dis)Similarity Effects x Gender on Self-Reported Victimization*.

|  | T2 |  | T3 |  |
| --- | --- | --- | --- | --- |
| Characteristics at baseline  (T1: October) | *b* | 95% *CI* | *b* | 95% *CI* |
| Friendships |  |  |  |  |
| *Person-fit/complete model* |  |  |  |  |
| Person score *x* | -0.23 | -1.31; 0.86 | 0.02 | -1.11; 1.16 |
| Person-group similarity *i* | **-1.91** | -3.38; -0.43 | **-2.15** | -3.74; -0.57 |
| Group-group similarity *i’* | *n/a* | *n/a* | 2.82 | 0.60; 2.26 |
| Person score *x**gender | -1.20 | -2.45; 0.05 | -0.24 | -1.39; 0.91 |
| Person-group similarity *i**gender | 1.23 | -0.39; 2.84 | 0.65 | -0.97; 2.26 |
| Social media connectedness |  |  |  |  |
| *Person-fit model* |  |  |  |  |
| Person score *x* | *n/a* | *n/a* | 0.83 | -0.14; 1.81 |
| Person-group similarity *i* | *n/a* | *n/a* | **-2.15** | -4.00; -0.31 |
| Person score *x**gender | *n/a* | *n/a* | 0.18 | -0.79; 1.14 |
| Person-group similarity *i**gender | *n/a* | *n/a* | 0.15 | -1.56; 1.87 |
| Social anxiety |  |  |  |  |
| *Person-fit model* |  |  |  |  |
| Person score *x* | 0.38 | -1.86; 2.63 | -0.18 | -2.25; 2.22 |
| Person-group similarity *i* | -0.44 | -3.95; 3.06 | -1.02 | -4.59; 2.55 |
| Person score *x**gender | -0.98 | -3.59; 1.62 | 0.35 | -2.40; 3.10 |
| Person-group similarity *i**gender | -0.49 | -5.35; 4.37 | 1.06 | -3.89; 6.01 |
| Disruptive behavior |  |  |  |  |
| *Person-fit model* |  |  |  |  |
| Person score *x* | -0.16 | -0.95; 0.62 | -0.81 | -1.53; -0.09 |
| Group-effect *x’* | 0.65 | -1.13; 2.44 | **-1.86** | -3.72; -0.00 |
| Person-group similarity *i* | **-1.15** | -2.37; 0.06 | **-2.43** | -3.54; -1.33 |
| Person score *x**gender | -0.61 | -1.72; 0.51 | 0.35 | -0.66; 1.36 |
| Group score *x'**gender | -0.62 | -2.02; 0.79 | **1.60** | 0.31; 2.89 |
| Person-group similarity *i**gender | 0.00 | -1.63; 1.63 | **1.57** | 0.06; 3.07 |

*Note.* Numbers in bold represent significant findings at the individual level.

SI 8. Traditional multilevel models

Additional analyses were performed using traditional multilevel analysis that included cross-level interactions between individual-level and classroom-level characteristics (Tables S12 and S13). With these models, we 1) first tested an omnibus interaction effect, and 2) then tested in follow-up analyses the main effect of *x* in contexts with a a) low-average norm, versus b) a high-average norm. We included aggregated standard deviations as covariates to control for homogeneity. For comprehensiveness we presented the follow-up results regardless of whether the omnibus interaction had a significant effect, for example because we had directional hypotheses for some characteristics and the omnibus effect might for that reason be non-significant. Further, in contrast to the other sensitivity analyses, for these analyses we also estimated effects on self-reported victimization because this approach was entirely different from the GAPIM approach used in the main analyses and sensitivity analyses.

Notably, the follow-up analyses are different from the GAPIM analyses because they can only present main effects of characteristics across low- versus high-average norm classrooms, instead of similarity effects. Moreover, they do not consider homogeneity between classroom members. Yet, the additional analyses point out consistent trends in the direction of person-group dissimilarity. For friendships and disruptive behaviors, similar to the GAPIM analyses, dissimilarity effects were most pronounced.

Specifically, for friendships, a consistent trend was shown in the sense that in classrooms with *higher* levels of friendships (with exceptions of bully-reported victimization on T2) stronger effects of having fewer friendships on victimization were observed. For social media connectedness, the effect on bully-reported victimization was in the same direction as in the GAPIM analyses: Especially in classrooms with *high* levels of social media connectedness, having fewer social media connections predicted victimization. For self-reported victimization however, especially in classrooms with *low* levels of social media connectedness (contexts that were not included in the GAPIM analyses, as these were directional in nature) having fewer social media connections predicted more victimization.

For disruptive behavior, all person x group-norm interactions were significant, except for effects on self-reported victimization T3, and the post-hoc analyses showed effects in the directions of dissimilarity; especially that acting disruptively in classrooms with *low* average levels of disruptive behaviors predicted more victimization. For social anxiety, despite an omnibus interaction effect on self-reported victimization T2, follow-up analyses did not show significant effects. Notably, in the GAPIM analyses the social anxiety effects were related to homogeneity of classroom norms - which could not be tested directly with the current analyses.

For pubertal development, similar to the GAPIM analyses, no (consistent) results were found.

Overall, results from traditional multilevel analyses were similar in the sense that they showed person-group dissimilarity effects of having fewer friendships than the peer-group norm on victimization, and of showing different levels of disruptive behaviors than the peer-group norm. For social media connectedness and social anxiety, mixed patterns were shown, which contrasted with the GAPIM analyses. The differences may be due to the different computations (for example, inclusion of the individual in computation of the classroom norm) and non-directional analyses (in the GAPIM analyses, in the directional analyses the low-friendship/social media connections and high-anxiety classrooms were not included), the different focus on main effects across contexts based on a cut-off instead of on similarity to the norm directly, and to the lack of potential to test interactions with homogeneity of the classroom norm – which was for example shown to be important for anxiety effects in the GAPIM analyses.

Table S12. *Fits of Traditional Multilevel Models: Person x Group-level Effects on Bully-Reported Victimization.*

|  | T2 (bully-rep) | |  | T3 (bully-rep) | |  | T2 (self-rep) | |  | T3 (self-rep) | |
| --- | --- | --- | --- | --- | --- | --- | --- | --- | --- | --- | --- |
| Characteristics at baseline (T1: October) | *AIC* | *BIC* |  | *AIC* | *BIC* |  | *AIC* | *BIC* |  | *AIC* | *BIC* |
| Friendships | 1190.4 | 1246.9 |  | 1150.6 | 1207.1 |  | 2153.6 | 2209.6 |  | 2001.2 | 2057.3 |
| Social media connectedness | n/a | n/a |  | 1141.3 | 1197.5 |  | n/a | n/a |  | 2036.7 | 2092.5 |
| Social anxiety | 1206.6 | 1262.8 |  | 1154.7 | 1210.8 |  | 1946.9 | 2002.7 |  | 2100.1 | 2144.8 |
| Disruptive behaviors | 1242.8 | 1299.4 |  | 1174.8 | 1231.4 |  | 2013.6 | 2069.7 |  | 2165.7 | 2221.7 |
| Boys’ pubertal development | 694.6 | 738.8 |  | 628.7 | 672.8 |  | 1067.6 | 1111.3 |  | 1147.6 | 1191.2 |
| Girls’ pubertal development | 496.2 | 540.6 |  | 536.1 | 580.3 |  | 858.9 | 903.0 |  | 883.0 | 926.9 |

AIC = Akaike Information Criterion. BIC = Bayesian Information Criterion.

Table S13. *Poisson Estimations of Traditional Multilevel Models of Individual x Group Effects on Victimization.*

|  | Bully-reported | | | | Self-reported | | | |
| --- | --- | --- | --- | --- | --- | --- | --- | --- |
|  | T2 |  | T3 |  | T2 |  | T3 |  |
| Characteristics at baseline (T1: October) | *b* | 95% *CI* | *b* | 95% *CI* | *b* | 95% *CI* | *b* | 95% *CI* |
| Friendships |  |  |  |  |  |  |  |  |
| Person score *x* | -1.67 | -2.44; -0.91 | -1.83 | -2.59; -1.07 | -2.04 | -2.73; -1.34 | -0.83 | -1.53; -0.12 |
| Norm *x̅* | 1.72 | -0.08; 3.52 | 1.49 | -0.36; 3.34 | -1.26 | -2.99; 0.47 | -0.45 | -2.65; 1.75 |
| Person x norm *x*x̅* | 0.53 | -1.48; 2.54 | -0.93 | -3.05; 1.19 | **-2.91** | -4.63; -1.19 | -0.36 | -2.24; 1.54 |
| Post-hoc |  |  |  |  |  |  |  |  |
| *x* if norm *x̅ =* low | **-2.02** | -2.66; -1.37 | **-1.39** | -2.04; -0.74 | **-0.90** | -1.33; -0.47 | -0.34 | -0.81; 0.13 |
| ***x* if norm  *x̅ =*  high** | **-1.40** | -2.11; -0.70 | **-1.61** | -2.33; -0.90 | **-1.41** | -2.05; -0.77 | **-1.30** | -1.83; -0.76 |
| Social media connectedness |  |  |  |  |  |  |  |  |
| Person score *x* | n/a | n/a | 0.13 | -0.44; 0.70 | n/a | n/a | -0.96 | -1.39; -0.52 |
| Norm *x̅* | n/a | n/a | 1.45 | 0.49; 2.41 | n/a | n/a | -0.45 | -1.42; 0.52 |
| Person x norm *x*x̅* | n/a | n/a | -0.17 | -0.48; 0.15 | n/a | n/a | **0.53** | 0.27; 0.80 |
| Post-hoc |  |  |  |  |  |  |  |  |
| *x* if norm *x̅ =* low | n/a | n/a | 0.08 | -0.17; 0.33 | n/a | n/a | **-0.33** | -0.50; -0.16 |
| ***x* if norm  *x̅ =*  high** | n/a | n/a | **-0.22** | -0.36; -0.08 | n/a | n/a | 0.00 | -0.13; 0.13 |
| Social anxiety |  |  |  |  |  |  |  |  |
| Person score *x* | -1.10 | -5.91; 3.71 | -0.28 | -4.70; 4.14 | **5.06** | 0.99; 9.12 | 3.60 | 1.29; 16.41 |
| Norm *x̅* | 0.06 | -6.63; 6.74 | -1.53 | -8.20; 5.13 | 11.40 | 5.58; 17.2 | 8.85 | 1.29; 16.41 |
| Person x norm *x*x̅* | -1.96 | -9.14; 5.22 | -0.80 | -7.36; 5.76 | **7.92** | 1.60; 14.23 | 5.40 | -1.79; 12.60 |
| Post-hoc |  |  |  |  |  |  |  |  |
| ***x* if norm  *x̅ =*  low** | 0.53 | -0.32; 1.38 | 0.62 | -0.25; 1.49 | 0.10 | -0.71; 0.92 | -0.02 | -0.89; 0.89 |
| *x* if norm *x̅ =* high | 0.08 | -0.44; 0.61 | 0.06 | -0.51; 0.64 | 0.03 | -0.40; 0.46 | 0.23 | -0.22; 0.68 |
| Disruptive behaviors |  |  |  |  |  |  |  |  |
| Person score *x* | **-4.98** | -8.72; -1.24 | **-4.16** | -6.85; -1.48 | **-2.87** | -5.31; -0.43 | 0.57 | -2.36; 3.51 |
| Norm *x̅* | -3.87 | -9.29; 1.56 | **-5.98** | -11.08; -0.88 | **-6.97** | -11.77; -2.17 | -3.69 | -9.76; 2.38 |
| Person x norm *x*x̅* | **-6.70** | -11.35; -2.04 | **-6.12** | -9.52; -2.72 | **-4.66** | -7.68; -1.63 | 0.26 | -3.40; 3.92 |
| Post-hoc |  |  |  |  |  |  |  |  |
| *x* if norm  *x̅ =*  low | **0.65** | 0.09; 1.20 | **1.15** | 0.54; 1.75 | **1.17** | 0.81; 1.52 | 0.21 | -0.27; 0.70 |
| *x* if norm *x̅ =* high | -0.59 | -1.40; 0.23 | -0.37 | -0.99; 0.25 | -0.18 | -0.81; 0.44 | 0.48 | -0.11; 1.07 |
| Pubertal development |  |  |  |  |  |  |  |  |
| Boys |  |  |  |  |  |  |  |  |
| Person score *x* | -0.11 | -0.41; 0.18 | -0.43 | -0.74; -0.11 | -0.15 | -0.38; 0.09 | -0.22 | -0.46; 0.03 |
| Norm *x̅* | 0.96 | -0.52; 2.45 | 0.49 | -1.02; 1.99 | 0.37 | -1.13; 1.88 | -0.78 | -2.93; 1.37 |
| Person x norm *x*x̅* | -0.21 | -1.02; 0.60 | -0.65 | -1.62; 0.32 | **0.66** | 0.03; 1.28 | 0.64 | -0.01; 1.30 |
| Post-hoc |  |  |  |  |  |  |  |  |
| *x* if norm  *x̅ =*  low | 0.37 | -0.19; 0.94 | -0.12 | -0.76; 0.51 | **-0.56** | -1.05; -0.07 | **-0.52** | -0.93; -0.11 |
| *x* if norm *x̅ =* high | -0.24 | -0.57; 0.06 | **-0.52** | -0.87; -0.19 | -0.00 | -0.25; 0.25 | -0.03 | -0.34; 0.27 |
| Girls |  |  |  |  |  |  |  |  |
| Person score *x* | 0.74 | -2.35; 3.83 | -0.65 | -3.32; 2.03 | -0.66 | -2.43; 1.11 | -0.05 | -1.83; 1.73 |
| Norm *x̅* | 2.67 | -0.84; 6.18 | -0.80 | -3.83; 2.24 | -0.69 | -2.90; 1.52 | 1.00 | -1.33; 3.33 |
| Person x norm *x*x̅* | -0.22 | -1.37; 0.94 | 0.19 | -0.84; 1.21 | 0.20 | -0.50; 0.91 | -0.02 | -0.75; 0.71 |
| Post-hoc |  |  |  |  |  |  |  |  |
| *x* if norm  *x̅ =*  low | 0.47 | -0.23; 1.15 | -0.14 | -0.71; 0.44 | -0.22 | -0.56; 0.12 | -0.15 | -0.46; 0.17 |
| *x* if norm *x̅ =* high | 0.17 | -0.22; 0.58 | -0.17 | -0.59; 0.25 | -0.07 | -0.40; 0.27 | -0.07 | -0.46; 0.32 |

*Note.* Numbers in bold represent significant findings.

SI 9. Analyses with reciprocal measure of friendship

We tested person-group dissimilarity effects for reciprocal friendships, in addition to the unilateral (received nominations) measure in the main analyses (see Tables S14-S17). For the short-term effects, the complete model fitted the data best and showed a significant negative person-group similarity effect in the short term (*IRR =* 0.18). For the long-term, the person-fit model fitted best and the other models also did not show additional significant effects.

For self-reported victimization, which was only used in the sensitivity analyses (see Tables S4 and 5), the short-term effects model showed similar fits for the person-fit and contrast interaction model, and the contrast interaction model also included additional significant effects. For the person-fit model, there was no significant similarity effect, but follow-up analyses of the contrast interaction model showed that in *homogeneous* classrooms there was a significant negative person-group similarity effect, *b*_i_ = -1.33, 95% *CI* = -2.27; -0.39; *IRR* = 0.26, and not in heterogeneous classrooms, *b*_i_ = 0.18, 95% *CI* = -1.97; 2.33; *IRR* = 1.20. The long-term effects showed no significant negative person-group similarity effects or other effects (*IRR =* 0.85).

Altogether, a measure of reciprocal instead of unilateral (received nominations for) friendships shows person-group dissimilarity effects similar to the main analyses. However, the long-term effect was non-significant, but an additional significant short-term effect for self-reported victimization as outcome was observed – especially in more homogeneous classrooms.

Table S14. *For Reciprocal Friendships: Comparison of Poisson GAPIM Submodels For Individual-Group (Dis)Similarity Effects on Bully-Reported Victimization.*

|  | T2 | | | | | |  | T3 | | | | | |
| --- | --- | --- | --- | --- | --- | --- | --- | --- | --- | --- | --- | --- | --- |
| Characteristic | Person-fit | | Complete | | Contrast interaction | |  | Person-fit | | Complete | | Contrast interaction | |
|  | *AIC* | *BIC* | *AIC* | *BIC* | *AIC* | *BIC* |  | *AIC* | *BIC* | *AIC* | *BIC* | *AIC* | *BIC* |
| Reciprocal friendships | 689.7 | 734.6 | **689.5** | **738.9** | 690.6 | 744.4 |  | **677.7** | **722.5** | 678.4 | 427.7 | 679.6 | 733.3 |

Table S15. *GAPIM Submodels: Poisson Estimations of Individual-Group (Dis)Similarity Effects of Reciprocal Friendships on Bully-Reported Victimization*.

|  | T2 |  | T3 |  |
| --- | --- | --- | --- | --- |
| Characteristics at baseline (T1: October) | *b* | 95% *CI* | *b* | 95% *CI* |
| Reciprocal friendships |  |  |  |  |
| *Complete model* |  |  |  |  |
| Person score *x* | 0.11 | -1.21; 1.42 | -1.22 | -2.49; 0.05 |
| Person-group similarity *i* | **-1.72** | -3.17; -0.29 | -0.47 | -1.82; 0.90 |
| Group-group similarity *i’* | 1.55 | -0.44; 3.54 |  |  |

*Note.* Numbers in bold represent significant findings. Directional analyses were conducted: the effect was only estimated among individuals who scored *≤* than the classroom norm *x’*. The group score *x’* did not improve the model and was excluded for parsimony.

Table S16. *For Reciprocal Friendships: Comparison of Poisson GAPIM Submodels For Individual-Group (Dis)Similarity Effects on Self-Reported Victimization.*

|  | T2 self-report | | | | | |  | T3 self-report | | | | | |
| --- | --- | --- | --- | --- | --- | --- | --- | --- | --- | --- | --- | --- | --- |
| Characteristic | Person-fit | | Complete | | Contrast interaction | |  | Person-fit | | Complete | | Contrast interaction | |
|  | *AIC* | *BIC* | *AIC* | *BIC* | *AIC* | *BIC* |  | *AIC* | *BIC* | *AIC* | *BIC* | *AIC* | *BIC* |
| Reciprocal friendships | 1046.2 | 1090.8 | 1047.3 | 1096.3 | **1039.6** | **1093.0** |  | **1128.2** | **1172.6** | 1130.2 | 1179.1 | 1130.7 | 1184.0 |

Table S17. *GAPIM Submodels: Poisson Estimations of Individual-Group (Dis)Similarity Effects of Reciprocal Friendships on Self-Reported Victimization*.

|  | T2 |  | T3 |  |
| --- | --- | --- | --- | --- |
| Characteristics at baseline (T1: October) | *b* | 95%CI | *b* | 95%CI |
| Reciprocal friendships |  |  |  |  |
| *Person-fit model* |  |  |  |  |
| Person score *x* | **-1.70** | -2.99; -0.43 | -0.31 | -1.56; 0.93 |
| Person-group similarity *i* | -0.35 | -1.80; 1.00 | -0.16 | -1.57; 1.24 |
| *Contrast interaction model* |  |  |  |  |
| Person score *x* | -0.52 | -2.10; 1.06 |  |  |
| Person-group similarity *i* | **-4.17** | -6.89; -1.45 |  |  |
| Group-group similarity *i’* | 0.65 | -2.06; 3.36 |  |  |
| Contrast interaction (*i*i’*) | **9.86** | 3.46; 16.3 |  |  |

*Note.* Numbers in bold represent significant findings at the individual level. Directional analyses were conducted: the effect was only estimated among individuals who scored *≤* than the classroom norm *x’*. The group score *x’* did not improve the models and was excluded for parsimony.

SI 10. Directional (non-hypothesized) analyses

Additional directional analyses were conducted to test whether the omnibus main analyses (i.e., analyses with effects in both directions) did not obscure important effects in one direction when, for example, the effect in the other direction was non-significant or in the reverse direction. These analyses were the same as those in the main analyses for relational characteristics and social anxiety, so testing the models only in the sample of those who scored, for example, below their descriptive classroom norm for the particular predictor. For the sake of completeness, we also report the results that were already reported in the main results of the directional analyses.

For relational characteristics, results showed that dissimilarity in friendships only predicted victimization for those who had fewer friends than the norm, and not for those who had more friends. For social media connections, there was a trend showing that more *similarity* predicted victimization for those who had more social media connections than the norm, but this was not a significant result.

For social anxiety, similarly, results showed that there were no associations between dissimilarity and victimization for those who were less socially anxious than peers.

For disruptive behaviors, like the main results, results from person-fit models showed that in the short term, dissimilarity predicted victimization for those who were less disruptive than the norm, while in the long term, dissimilarity predicted victimization for those who were more disruptive than the norm. Additionally, in the current analyses, a contrast interaction effect was again found for the short-term model, but only for those who acted more disruptive than the norm. However, follow-up analyses in homogeneous (*b_i_* = -0.49, 95% *CI* = -4.24, 3.26, *IRR* = 0.24) and heterogeneous classrooms (*b_i_* = -0.20, 95% *CI* = -2.69, 2.29, *IRR* = 0.82) did not show any effects of dissimilarity and were not reliable because of the low number of victims (*N <* 30) in the homogeneous sample.

For pubertal development, for boys, no individual dissimilarity effects were found, but for those who were advanced than the norm in the short term and those who were less advanced in the long term, more homogeneity in pubertal development (*i*’) predicted more victimization. For girls, the only difference with the main analyses – in which no dissimilarity effects were found – was that being more *similar* predicted victimization in the short term for more advanced girls than the norm among their female classmates. Being less advanced, when other girls are on average less advanced too, predicted victimization. However, this result should be interpreted with caution because it is not shown in the other analyses and not consistent over time.

Table S18. *Two Directions Separately (Short-term Effects): Comparison of Poisson GAPIM Submodels For Individual-Group (Dis)Similarity Effects on Bully-Reported Victimization.*

|  | T2 | | | | | | | | | | | | |
| --- | --- | --- | --- | --- | --- | --- | --- | --- | --- | --- | --- | --- | --- |
|  | Individual < norm | | | | | |  | Individual > norm | | | | | |
| Predictor at baseline  (T1: October) | Person-fit | | Complete | | Contrast interaction | |  | Person-fit | | Complete | | Contrast interaction | |
|  | AIC | BIC | AIC | BIC | AIC | BIC |  | AIC | BIC | AIC | BIC | AIC | BIC |
| Friendships | **782.4** | **826.5** | 784.3 | 832.8 | 785.1 | 837.9 |  | **385.9** | **430.7** | 387.8 | 437.1 | 389.4 | 443.2 |
| Social media  connectedness |  |  |  |  |  |  |  |  |  |  |  |  |  |
| Social  anxiety | **699.8** | **745.3** | 698.1 | 748.2 | 699.63 | 754.2503 |  | **496.8** | **539.4** | 495.5 | 542.3 | 497.4 | 548.5 |
| Disruptive behaviors | **904.3** | **947.5** | 894.3 | 942.4 | 894.7 | 947.5 |  | **428.6** | **463.6** | 425.5 | 464.3 | **422.4** | **465.2** |
| Boys’ pubertal  development | **387.4** | **421.1** | 388.9 | 426.3 | 389.4 | 430.6 |  | **310.5** | **343.8** | 308.2 | 345.1 | 307.3 | 347.9 |
| Girls’  pubertal development | **203.9** | **236.9** | 205.5 | 242.1 | 206.7 | 247.0 |  | **313.1** | **347.2** | 314.0 | 353.0 | 316.8 | 358.6 |

Table S19. *Two Directions Separately (Long-Term Effects): Comparison of Poisson GAPIM Submodels For Individual-Group (Dis)Similarity Effects on Bully-Reported Victimization.*

|  | T3 | | | | | | | | | | | | |
| --- | --- | --- | --- | --- | --- | --- | --- | --- | --- | --- | --- | --- | --- |
|  | Individual < norm | | | | | |  | Individual > norm | | | | | |
| Predictor at baseline  (T1: October) | Person-fit | | Complete | | Contrast interaction | |  | Person-fit | | Complete | | Contrast interaction | |
|  | AIC | BIC | AIC | BIC | AIC | BIC |  | AIC | BIC | AIC | BIC | AIC | BIC |
| Friendships | 735.0 | 779.0 | **731.6** | **778.0** | 733.1 | 785.8 |  | **434.6** | **479.4** | 436.6 | 485.9 | 438.0 | 491.7 |
| Social media  connectedness | **616.9** | **661.0** | 618.3 | 666.9 | 620.3 | 673.3 |  | **528.1** | **571.9** | 529.7 | 577.8 | 531.5 | 584.1 |
| Social  anxiety | **717.7** | **763.2** | 719.7 | 769.7 | 721.4 | 776.0 |  | **445.7** | **488.2** | 447.6 | 494.3 | 449.1 | 500.1 |
| Disruptive behaviors | **733.1** | **781.1** | 734.9 | 787.7 | 736.5 | 794.1 |  | **445.9** | **484.7** | 447.0 | 489.7 | 447.6 | 494.2 |
| Boys’ pubertal  development | 386.6 | 420.2 | **384.7** | **422.1** | 386.1 | 427.3 |  | 271.1 | 304.3 | **263.9** | **300.8** | 263.6 | 304.1 |
| Girls’  pubertal development | **279.4** | **312.3** | 281.3 | 317.9 | 283.3 | 323.6 |  | **276.0** | **310.2** | 277.7 | 315.6 | 279.5 | 321.3 |

Table S20. *GAPIM Directional Submodels: Poisson Estimations of Individual-Group (Dis)Similarity Effects on Bully-Reported Victimization*.

|  | T2 | | | |  | T3 | | | |
| --- | --- | --- | --- | --- | --- | --- | --- | --- | --- |
|  | Individual < norm | | Individual > norm | |  | Individual < norm | | Individual > norm | |
| Characteristics at baseline (T1: October) | *b* | *95% CI* | *b* | *95% CI* |  | *b* | *95% CI* | *b* | *95% CI* |
| Friendships |  |  |  |  |  |  |  |  |  |
| *Person-fit/complete model* |  |  |  |  |  |  |  |  |  |
| Person score *x* | -0.89 | -1.79; 0.01 | 0.08 | -2.18; 2.35 |  | -0.13 | -1.07; 0.80 | 0.78 | -0.80; 2.36 |
| Person-group similarity *i* | -1.27 | -2.45; -0.11 | 0.83 | -2.50; 4.16 |  | **-1.74** | -2.95; -0.54 | 1.67 | -0.78; 4.11 |
| Person-group similarity *i'* |  |  |  |  |  | **2.74** | 0.54; 4.94 |  |  |
| Social media connections |  |  |  |  |  |  |  |  |  |
| *Person-fit model* |  |  |  |  |  |  |  |  |  |
| Person score *x* | *n/a* | *n/a* | *n/a* | *n/a* |  | **0.92** | 0.16; 1.68 | 0.76 | -0.15; 1.67 |
| Person-group similarity *i* | *n/a* | *n/a* | *n/a* | *n/a* |  | **-2.03** | -3.44; -0.62 | 1.50^1^ | 0.01; 2.99 |
| Social anxiety |  |  |  |  |  |  |  |  |  |
| *Person-fit model* |  |  |  |  |  |  |  |  |  |
| Person score *x* | -0.08 | -1.11; 0.95 | -0.22 | -2.07; 1.63 |  | -0.16 | -1.15; 0.85 | 0.10 | -1.85; 2.04 |
| Person-group similarity *i* | -0.48 | -2.34; 1.39 | -0.58 | -3.49; 2.32 |  | 2.93 | -2.19; 1.51 | -0.62 | -3.72; 2.48 |
| Disruptive behaviors |  |  |  |  |  |  |  |  |  |
| *Person-fit/complete model* |  |  |  |  |  |  |  |  |  |
| Person score *x* | 0.88 | -0.11; 1.87 | -0.92 | -2.16; 0.32 |  | -0.06 | -1.25; 1.13 | -1.07 | -2.19; 0.05 |
| Person-group similarity *i* | **-1.14** | -2.09; -0.19 | -1.75^2^ | -3.65; 0.15 |  | -0.72 | -2.31; 0.88 | **-1.84** | -3.60; -0.10 |
| Group-group similarity *i’* |  |  |  |  |  |  |  |  |  |
| *Contrast interaction model* |  |  |  |  |  |  |  |  |  |
| Person score *x* |  |  | 0.64 | -0.96; 2.24 |  |  |  |  |  |
| Person-group similarity *i* |  |  | 2.28 | -1.13; 5.68 |  |  |  |  |  |
| Group-group similarity *i’* |  |  | 0.78 | -0.52; 2.08 |  |  |  |  |  |
| Contrast interaction (*i*i’*) |  |  | **-3.44** | -6.39; -0.49 |  |  |  |  |  |
| Pubertal development |  |  |  |  |  |  |  |  |  |
| Boys |  |  |  |  |  |  |  |  |  |
| *Person-fit/complete model* |  |  |  |  |  |  |  |  |  |
| Person score *x* | 0.87 | -1.54; 3.28 | -1.44 | -4.04; 1.15 |  | -0.51 | -2.66; 1.65 | -0.48 | -3.21; 2.24 |
| Person-group similarity *i* | -1.72 | -4.86; 1.42 | -0.29 | -3.50; 2.92 |  | -0.40 | -3.21; 2.41 | -0.21 | -3.55; 3.51 |
| Group-group similarity *i’* |  |  | **6.69** | 2.64; 10.74 |  | **2.99** | 0.06; 5.92 |  |  |
| Girls |  |  |  |  |  |  |  |  |  |
| *Person-fit model* |  |  |  |  |  |  |  |  |  |
| Person score *x* | 1.19 | -1.16; 3.55 | **3.64** | 1.43; 5.84 |  | -1.11 | -2.90; 0.67 | 1.38 | -1.21; 3.98 |
| Group score *x’* | -1.76 | -4.97; 1.45 | **4.25** | 1.68; 6.82 |  | 1.01 | -1.14; 3.18 | 1.19 | -2.19; 4.57 |

*Note.* Numbers in bold represent significant findings at the individual level. ^1^ *p* = .048, not significant when applying the FDR method. ^2^ *p* = .07.

SI 11. Analyses using a product instead of absolute difference term for dissimilarity

In the main analyses, we selected the least restrictive method to calculate similarity (square root of absolute difference) due to our small sample of victims, *N* = 138 (Kenny, 2021). The models were additionally tested using a more stringent definition of moderation, by calculating the product term (*x*x’*) between the individual and group norm variables (Tables S21 and S22) instead of the square root of the absolute difference (see the manuscript for full formulas).

Regarding friendships, for the short-term effect of friendships, the person-fit model fitted the data best and the other models did not show significant group-group similarity effects or a contrast interaction effect. The person-fit model showed no significant person-group similarity effect (*IRR =* 1.67). For the long-term effect, the complete model again fitted best but showed only a trend for a negative person-group similarity effect (*IRR* = 0.35, *p = .*098) when others were more similar to each other.

Regarding social media connections, similar to the main analyses, the person-fit model fitted the data best and the other models did not show significant group-group similarity effects or a contrast interaction effect. The person-fit model showed a significant negative person-group similarity effect (*IRR =* 0.19) for those who had fewer social media connections then the descriptive classroom norm.

Regarding disruptive behaviors, for the short-term effects, the person-fit model showed a significant negative person-group similarity effect (*IRR =* 0.16) and fitted similar to the contrast interaction model (*IRR =* 0.06). The person-fit model showed that being more dissimilar in individual disruptive behaviors to the peer group-norm predicted more victimization. Follow-up analyses replicated the main analyses in showing that being dissimilar in terms of disruptive behaviors predicted victimization in terms of acting *less* disruptively than the peer-group norm, *b*_i_ = -2.16, 95% *CI* = -3.26; -1.07, *IRR =* 0.11*,* and not in terms of acting *more* disruptively, *b*_i_ = -1.69, 95% *CI* = -3.50; 0.11, *IRR =* 0.18; *p =* .07, although the latter was a trend effect, similar to the main analyses.

Further, similar to the main analyses, the contrast interaction model additionally showed that more dissimilarity for those who acted less disruptive than the norm especially predicted victimization in *more* *homogeneous* classrooms (*b* = -4.96, 95% *CI* = -7.15; -2.78, *IRR =* 0.01) and not in heterogeneous classrooms (*b* = -2.80, 95% *CI* = -6.56; 0.96, *IRR =* 0.06). Again, there were no person-group similarity effects of acting more disruptively in either homogeneous (*b* = -2.03, 95% *CI* = -1.58; 5.64, *IRR =* 0.28) or heterogeneous (*b* = -1.28, 95% *CI* = -4.18; 1.60, *IRR =* 7.62).

For the long-term, the person-fit model fitted best (*IRR =* 0.20) and the other models also showed no additional effects. Again similar to the main analyses, being more dissimilar in individual disruptive behaviors to the peer group-norm predicted more victimization. Follow-up analyses replicated the main analyses in showing that being dissimilar in terms of disruptive behaviors predicted victimization in terms of acting *more* disruptively than the peer-group norm, *b*_i_ = -1.67, 95% *CI* = -3.29; -0.07, *IRR =* 0.19*,* and not in terms of acting *less* disruptively, *b*_i_ = 0.02, 95% *CI* = -1.25; 1.29, *IRR =* 1.18.

For pubertal development, for boys the person-fit model fitted the data best in the short term (*IRR =* 0.34) and the complete model fitted the data best in the long term (*IRR* = 0.22). Both showed no significant person-group dissimilarity effects, but a group-group score showing that in more homogeneous classrooms regarding pubertal development, individuals were more likely to be victimized. For girls, the person-fit models fitted the data best but showed no significant person-group similarity effects (short-term *IRR* = 0.67, long-term *IRR* = 1.53) and the other models also showed no additional significant effects. The only effect was that when the group norm was that girls were on average more advanced in their pubertal development, individual victimization was higher in the short term.

In conclusion, using a more stringent operationalization of person-group dissimilarity showed similar results, with the exception that the short-term effect of person-group dissimilarity among those with fewer friendships than the norm became non-significant and the long-term effect only showed a trend. This may be due to the analyses being underpowered because of the small number of victims in some analyses.

Table S21. *Based on Product Terms: Comparison of Poisson GAPIM Submodels For Individual-Group (Dis)Similarity Effects on Bully-Reported Victimization.*

|  | T2 | | | | | |  | T3 | | | | | |
| --- | --- | --- | --- | --- | --- | --- | --- | --- | --- | --- | --- | --- | --- |
| Predictor at baseline  (T1: October) | Person-fit | | Complete | | Contrast interaction | |  | Person-fit | | Complete | | Contrast interaction | |
|  | *AIC* | *BIC* | *AIC* | *BIC* | *AIC* | *BIC* |  | *AIC* | *BIC* | *AIC* | *BIC* | *AIC* | *BIC* |
| Friendships | **785.7** | **829.8** | 787.2 | 835.6 | 787.5 | 840.3 |  | 737.9 | 781.8 | **735.9** | **784.2** | 737.2 | 789.9 |
| Social media  connectedness |  |  |  |  |  |  |  | **618.9** | **663.1** | 620.5 | 669.1 | 622.5 | 575.5 |
| Social  anxiety | **385.2** | **430.0** | 387.2 | 436.4 | 388.8 | 442.6 |  | **436.3** | **481.2** | 438.2 | 487.4 | 440.2 | 493.9 |
| Disruptive behaviors | **1237.0** | **1293.5** | 1235.6 | 1297.4 | **1233.7** | **1300.6** |  | **1175.5** | **1232.0** | 1177.4 | 1239.1 | 1179.4 | 1246.2 |
| Boys’ pubertal  development | **690.5** | **734.6** | 689.4 | 738.0 | 691.1 | 744.1 |  | 633.3 | 677.4 | **626.3** | **674.8** | 627.3 | 680.2 |
| Girls’  pubertal development | **490.5** | **534.8** | 491.8 | 540.6 | 491.5 | 544.7 |  | **535.2** | **579.6** | 536.2 | 584.9 | 538.0 | 591.1 |

AIC = Akaike Information Criterion. BIC = Bayesian Information Criterion. Numbers in bold refer to the final model(s), which were multiple models if Δ BIC<10 and an additional effect (of *i'* in the complete model, or of *i*i'* in the contrast interaction model) was observed.
Table S22. *GAPIM Product Submodels: Poisson Estimations of Individual-Group (Dis)Similarity Effects on Bully-Reported Victimization*.

|  | T2 | | T3 | |
| --- | --- | --- | --- | --- |
| Characteristics at baseline (T1: October) | *b* | *95% CI* | *b* | *95% CI* |
| *Directional analyses^1^* | | | | |
| Friendships |  |  |  |  |
| *Person-fit/complete model* |  |  |  |  |
| Person score *x* | -1.55 | -2.18; -0.90 | **-1.25** | -1.90; -0.61 |
| Person-group similarity *i* | 0.51 | -1.14; 2.16 | -1.37^2^ | -2.99; 0.25 |
| Person-group similarity *i'* |  |  | **2.69** | 0.14; 5.24 |
| Social media connections |  |  |  |  |
| *Person-fit model* |  |  |  |  |
| Person score *x* | *n/a* | *n/a* | 0.46 | -0.11; 1.03 |
| Person-group similarity *i* | *n/a* | *n/a* | **-1.66** | -3.03; -0.32 |
| Social anxiety |  |  |  |  |
| *Person-fit model* |  |  |  |  |
| Person score *x* | -0.10 | -1.26; 1.07 | -0.05 | -1.10; 1.00 |
| Person-group similarity *i* | 1.73 | -1.73; 5.19 | 0.22 | -2.98; 3.43 |
| *Non-directional analyses* | | | | |
| Disruptive behaviors |  |  |  |  |
| *Person-fit/complete model* |  |  |  |  |
| Person score *x* | **-1.00** | -1.83; -0.17 | **-0.70** | -1.30; -0.10 |
| Group score *x’* | -0.33 | -2.03; 1.37 | -1.28 | -3.02; 0.46 |
| Person-group similarity *i* | **-1.85** | -3.05; -0.65 | **-1.59** | -2.47; -0.71 |
| Group-group similarity *i’* |  |  |  |  |
| *Contrast interaction model* |  |  |  |  |
| Person score *x* | -0.32 | -1.35; 0.70 |  |  |
| Person-group similarity *i* | 2.46 | -0.30; 5.21 |  |  |
| Group-group similarity *i’* | **3.31** | 0.50; 6.11 |  |  |
| Contrast interaction (*i*i’*) | **-2.85** | -5.65; -0.05 |  |  |
| Pubertal development |  |  |  |  |
| Boys |  |  |  |  |
| *Person-fit/complete model* |  |  |  |  |
| Person score *x* | -0.27 | -0.98; 0.44 | **-0.99** | -1.82; -0.16 |
| Group score *x* | 1.70 | -0.86; 4.27 | 1.05 | -1.55; 3.65 |
| Person-group similarity *i* | -1.08 | -3.76; 1.60 | -1.45 | -4.54; 1.64 |
| Group-group similarity *i’* |  |  | **4.59** | 1.61; 7.75 |
| Girls |  |  |  |  |
| *Person-fit model* |  |  |  |  |
| Person score *x* | 0.71 | 0.05; 1.36 | -0.37 | -0.95; 0.21 |
| Group score *x’* | **3.01** | 0.57; 5.46 | -0.70 | -3.15; 1.75 |
| Person-group similarity *i* | -0.40 | -3.32; 2.52 | 0.43 | -2.38; 3.23 |

*Note.* Numbers in bold represent significant findings at the individual level. ^1^Directional analyses were conducted by estimating the effect only among individuals who scored *≤* (friendships, social media connectedness) or *≥* (social anxiety) than the classroom norm *x’*. The group score *x’* did not improve the model and was excluded for parsimony. ^2^ *p* = .098.
